# Shear stress regulates endothelial cell autophagy via redox regulation and Sirt1 expression

**DOI:** 10.1038/cddis.2015.193

**Published:** 2015-07-16

**Authors:** J Liu, X Bi, T Chen, Q Zhang, S-X Wang, J-J Chiu, G-S Liu, Y Zhang, P Bu, F Jiang

**Affiliations:** 1 Key Laboratory of Cardiovascular Remodeling and Function Research, Qilu Hospital, Shandong University, Jinan, China; 2 Institute of Cellular and System Medicine, National Health Research Institutes, Zhunan, Taiwan; 3 Centre for Eye Research Australia, Department of Ophthalmology, University of Melbourne, East Melbourne, Victoria, Australia; 4 Department of Cardiology, Qilu Hospital, Shandong University, Jinan, China; 5 Department of Pathophysiology, Medical School, Shandong University, Jinan, China

## Abstract

Disturbed cell autophagy is found in various cardiovascular disease conditions. Biomechanical stimuli induced by laminar blood flow have important protective actions against the development of various vascular diseases. However, the impacts and underlying mechanisms of shear stress on the autophagic process in vascular endothelial cells (ECs) are not entirely understood. Here we investigated the impacts of shear stress on autophagy in human vascular ECs. We found that shear stress induced by laminar flow, but not that by oscillatory or low-magnitude flow, promoted autophagy. Time-course analysis and flow cessation experiments confirmed that this effect was not a transient adaptive stress response but appeared to be a sustained physiological action. Flow had no effect on the mammalian target of rapamycin-ULK pathway, whereas it significantly upregulated Sirt1 expression. Inhibition of Sirt1 blunted shear stress-induced autophagy. Overexpression of wild-type Sirt1, but not the deacetylase-dead mutant, was sufficient to induce autophagy in ECs. Using both of gain- and loss-of-function experiments, we showed that Sirt1-dependent activation of FoxO1 was critical in mediating shear stress-induced autophagy. Shear stress also induced deacetylation of Atg5 and Atg7. Moreover, shear stress-induced Sirt1 expression and autophagy were redox dependent, whereas Sirt1 might act as a redox-sensitive transducer mediating reactive oxygen species-elicited autophagy. Functionally, we demonstrated that flow-conditioned cells are more resistant to oxidant-induced cell injury, and this cytoprotective effect was abolished after inhibition of autophagy. In summary, these results suggest that Sirt1-mediated autophagy in ECs may be a novel mechanism by which laminar flow produces its vascular-protective actions.

Vascular endothelial cells (ECs) are fundamentally important in maintaining structural and functional homeostasis of blood vessels. Normal biological functions of ECs are highly sensitive to the biomechanical stimuli induced by blood flow, of which shear stress acting on the surface of EC has been recognized to be one of the most important vasoactive factors in EC.^1, 2^ A relatively high level of laminar shear stress is cytoprotective, whereas abnormal (low-magnitude or oscillatory) shear stress is a detrimental cellular stress to ECs.^1^ Transduction of the mechanical signals involves multiple messenger molecules and signaling proteins, which collectively regulate important endothelial functions, such as gene expression, proliferation, migration, morphogenesis, permeability, thrombogenicity, and inflammation.^2^

Autophagy (also known as macroautophagy) is an evolutionarily conserved cellular stress response.^3, 4^ Autophagy is a cellular self-digestion process, which is responsible for degradation of misfolded proteins and damaged organelles. Autophagic process is mainly mediated by the formation of autophagosome, a double-membrane vacuole structure containing engulfed cellular components. This process requires expression of a group of key genes involved in autophagy, including LC3A, beclin-1, Atg5, Atg7, and Atg12, for example.^3, 5^ Autophagosomes fuse with lysosomes, forming autolysosomes, where the cellular components are degraded by various hydrolases in an acidified environment.^4, 5^ In ECs, an autophagic response can be initiated by different stress stimuli.^6, 7, 8^ It is noted that the cellular outcome following autophagy induction in ECs varies depending on the nature of stimuli and specific experimental settings.^6, 7, 9, 10^ Moreover, there is evidence showing that autophagy may also be involved in modulating other EC functions such as angiogenesis and cellular senescence.^11, 12^ Therefore, understanding the regulatory mechanisms of autophagy in ECs will be important for discovery of strategies to protect normal endothelial functions. Recently, Guo *et al.* provided some evidence indicating that the autophagic process in EC might be affected by shear stress.^13^ This argument, however, was only based on observations of changed expression levels of LC3 and beclin-1; further experimental evidence is needed to confirm such an effect of shear stress on autophagy. More importantly, the mechanisms underlying this phenomenon are not understood. Different signaling pathways may be involved in modulating autophagy in ECs.^14, 15, 16^ For example, inhibition of the mTOR (mammalian target of rapamycin) pathway by rapamycin-induced endothelial autophagy and prevented energy stress-triggered cell damage.^16^ There is also evidence indicating a potential role of Sirt1.^14^ Moreover, accumulating evidence has suggested that reactive oxygen species (ROS) are closely implicated in modulating autophagic responses via complex interactions with other autophagy-related factors.^15^ Despite of these results, the signaling mechanisms of shear stress-regulated autophagy in EC remain to be defined. Hence, here we aim to delineate the impacts and underlying mechanisms of shear stress on autophagy in human vascular ECs.

## Results

### Laminar flow promotes autophagic response in ECs

**Figure 1:**
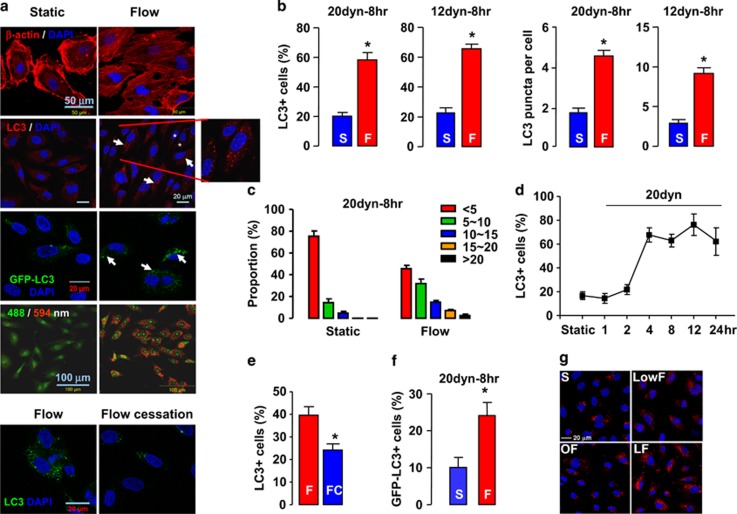
Laminar shear stress enhanced autophagic response in cultured endothelial cells. (**a**) Fluorescence images showing shear stress (20 dyn/cm^2^ for 8 h)-induced: (from top to bottom) realignment of actin fibers in human umbilical vein endothelial cells (HUVECs); accumulation of LC3 puncta (arrows) in HUVECs (asterisks indicate punctate LC3- cells); puncta accumulation of GFP-LC3 (arrows) in transfected telomerase-immortalized human microvascular endothelium cell (TIME) cells; acidification of autolysosomes detected with acridine orange staining (red fluorescence represents acidic vesicles); flow cessation decreased the abundance of GFP-LC3 in shear-adapted TIME cells. (**b**) The average number of LC3 puncta per cell and proportion of punctate LC3+ cells under static (S) and laminar flow (F) conditions. (**c**) Comparison of the proportion of cells containing different numbers of LC3 puncta under static and flow conditions. (**d**) Time course of laminar flow-induced autophagic response in HUVECs. (**e**) Effects of flow cessation (FC) on punctate LC3 in cells adapted to flow for 8 h. (**f**) Semi-quantitative data showing the effect of laminar flow on GFP-LC3 puncta accumulation in transfected TIME cells. (**g**) LC3 immunofluorescence showing that low-magnitude flow (LowF; 4 dyn/cm^2^) or oscillatory flow (OF; ±5 dyn/cm^2^ at 1 Hz) did not have the same effect as laminar flow (12 dyn/cm^2^) on autophagy induction. Data are mean±standard error of the mean (S.E.M.). *P<0.05, unpaired *t*-test, *n*=3–5. LF, laminar flow

**Figure 2:**
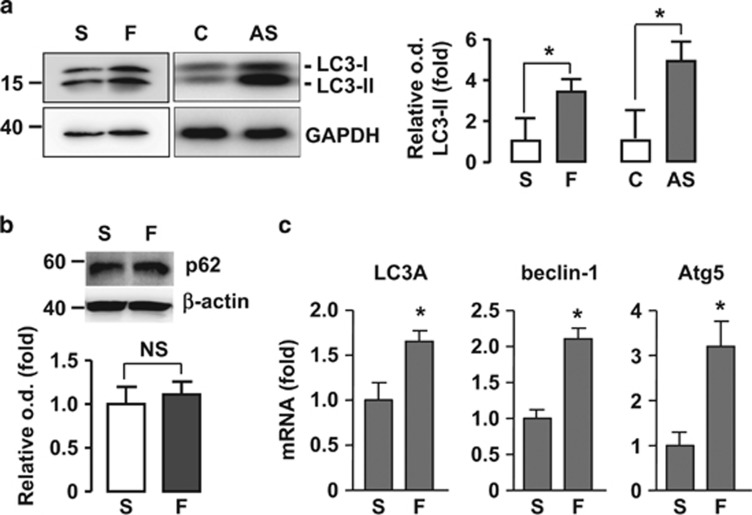
Effects of laminar flow (20 dyn/cm^2^) on (**a**) LC3 level, (**b**) p62 level, and (**c**) mRNA levels of LC3A, beclin-1, and Atg5. In **a**, autophagy induction by amino-acid starvation (by treating cells with Hank's balanced salt solution for 4 h) was used as a positive control. Data are mean±S.E.M. *P<0.05, unpaired *t*-test, *n*=3–4. C, control; S, static; F, flow; NS, no significance; AS, amino-acid starvation

To determine the effects of different types of shear stress on autophagic response in ECs, we treated human umbilical vein ECs (HUVECs) with laminar flow (12 or 20 dyn/cm^2^), oscillatory flow (±5 dyn/cm^2^ at 1 Hz), or low-magnitude flow (4 dyn/cm^2^). As shown in Figure 1a, application of laminar flow induced realignment of the actin fibers along the direction of flow. Using LC3 immunofluorescence, we showed that laminar flow significantly increased the abundance of LC3 puncta (Figures 1a–c), indicating an increased level of autophagy. The flow-induced autophagic response was comparable to that induced by amino-acid starvation (Supplementary Figure I and Figure 2a), which was used to mimic a positive control response. It should be noted that shear stress- and amino-acid starvation-induced responses involve distinct mechanisms (see below). To exclude the possibility that laminar flow-induced autophagy is just a transient adaptive response of static cells, we performed a time-course experiment and showed that the autophagic response was maintained at a stable level up to 24 h (Figure 1d). To exclude any artifacts introduced by LC3 immunostaining, we expressed ectopic GFP-LC3 in the human microvascular endothelium cell line TIME (telomerase-immortalized human microvascular endothelium) cells and then treated the cells with laminar flow. We demonstrated that flow induced similar GFP-LC3 puncta accumulation in TIME cells (Figures 1a and f). Then we performed a reversal experiment by withdrawing shear stimulus from flow-adapted cells. We showed that flow cessation for 4 h significantly decreased the abundance of LC3 puncta (Figures 1a and e). We also examined the time course of the effect of flow cessation on the autophagic response in HUVECs. We found that the abundance of LC3 puncta started to decline from 2 h after flow cessation, and returned to the basal level at 8 h (Supplementary Figure II). To determine whether pathological types of shear stress induced by oscillatory or low-magnitude flow had similar effects on endothelial autophagy, we treated HUVECs with oscillatory flow (by changing the direction of flow every half second with an amplitude of 5 dyn/cm^2^, i.e., ±5 dyn/cm^2^ at 1 Hz) and low-magnitude flow (4 dyn/cm^2^), and demonstrated that these types of flow failed to reproduce the stimulating effect of laminar flow on autophagy (Figure 1g and Supplementary Figure III).

Autophagic process results in acidification of autolysosomes and this can be monitored by acridine orange staining.^17^ We stained the cells with acridine orange and showed that laminar flow increased the amount of red fluorescence (Figure 1a). Next, we performed western blots for LC3. As shown in Figure 2a, flow significantly increased both of LC3-II and the amount of total LC3. In contrast, there was not a significant change in the protein level of p62 (Figure 2b). There is evidence showing that autophagy-inducing stimuli, especially oxidative stress, can upregulate the expression of p62.^18, 19^ Therefore, we measured the mRNA levels of p62. We found that shear stress enhanced the p62 mRNA expression by 2.9±0.9-fold (*P*<0.05). Hence, the unchanged p62 protein level after shear stress stimulation may indeed indicate an enhancement in the p62 protein clearance, supporting an increased autophagic flux rate. We also measured the mRNA levels of LC3A, beclin-1, and Atg5, which were all significantly upregulated by flow (Figure 2c). To verify that a basal level of autophagic flux was still present in the static cells used, we treated cells with the autophagy inhibitor chloroquine (50 *μ*M), and demonstrated that chloroquine induced significant accumulation of LC3 as shown in Supplementary Figure IV. To further confirm that the increase in LC3 puncta accumulation induced by shear stress was not due to impaired autophagic flux, we pretreated the cells with bafilomycin A1, an inhibitor of the late phase of autophagy. We showed that bafilomycin alone increased LC3 puncta, whereas application of laminar flow also exhibited an increasing effect on this response in the presence of bafilomycin (Supplementary Figure V).

### Sirt1 is essential in mediating shear stress-induced autophagy in EC

**Figure 3:**
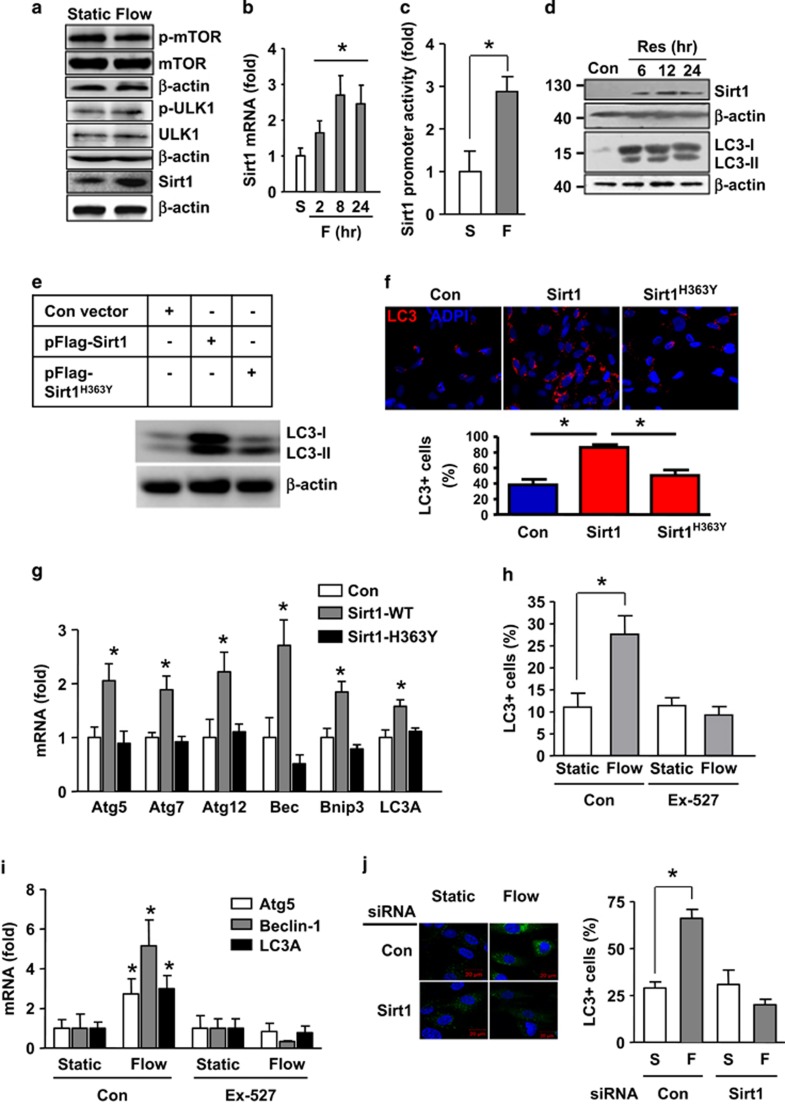
Sirt1 was critical in mediating shear stress-induced autophagy in endothelial cells. (**a**) Effects of laminar flow on the phosphorylation levels of mTOR and ULK1, and the expression level of Sirt1 (representative images from four experiments). (**b**) Time course of the effect of laminar flow on the mRNA expression of Sirt1. (**c**) Effect of laminar flow on the promoter activity of human Sirt1 as measured by luciferase reporter assay. (**d**) Effects of the Sirt1 activator resveratrol (10 *μ*M) on the protein levels of Sirt1 and LC3. (**e**) Effects of overexpression of Flag-tagged wild-type Sirt1 and Sirt1-H363Y mutant on LC3 expression. (**f**) Effects of Sirt1 and Sirt1-H363Y overexpression on accumulation of LC3 puncta. (**g**) Effects of wild-type Sirt1 and Sirt1-H363Y overexpression on the mRNA expression levels of various autophagy-related genes as indicated. (**h**) Effects of the Sirt1 inhibitor EX-527 (10 *μ*M) on flow-induced autophagy. (**i**) Effects of EX-527 on flow-induced upregulation of Atg5, beclin-1, and LC3A. (**j**) Effects of Sirt1 gene silencing with siRNA on flow-induced LC3 puncta accumulation. Data are mean±S.E.M. *P<0.05, unpaired *t*-test or one-way analysis of variance, *n*=3–4. Res, resveratrol

The mTOR-ULK pathway is the master regulator of autophagy. We therefore examined whether shear stress-induced autophagy involved the mTOR-ULK pathway. Western blot analysis showed that under current experimental conditions, laminar flow had no significant impacts on the phosphorylation levels of mTOR or ULK1 (Figure 3a and Supplementary Figure VI), suggesting that this pathway was unlikely to have a major role. As emerging evidence has suggested that the nicotinamide adenine dinucleotide (NAD^+^)-dependent protein deacetylase Sirt1 may have an important role in modulating autophagy. To examine this possibility, we measured Sirt1 expression in ECs. As shown in Figures 3a and b and Supplementary Figure VI, laminar flow significantly increased Sirt1 mRNA and protein levels. We also showed that flow significantly enhanced the Sirt1 promoter activity (Figure 3c), indicating that shear stress might directly regulate Sirt1 transcription in EC.

To clarify whether Sirt1 is necessary and sufficient in mediating the autophagic response in EC, we first treated the cells with the Sirt1-activating compound resveratrol, and demonstrated that resveratrol upregulated Sirt1 expression and triggered autophagy in EC (Figure 3d). Next, we performed Sirt1 gain-of-function experiments by overexpressing the wild-type human Sirt1; we found that Sirt1 overexpression significantly increased the level of LC3-II (3.9±0.3-fold, *P*<0.05, *n*=3) and accumulation of LC3 puncta (Figures 3e and f). In contrast, expression of the deacetylase-dead mutant Sirt1-H363Y had minor effects (Figures 3e and f). Moreover, we showed that the wild-type Sirt1, but not the H363Y mutant, significantly upregulated the expressions of Atg5, Atg7, Atg12, beclin-1, Bnip3, and LC3A (Figure 3g). To further confirm the role of Sirt1, we pretreated the cells with the specific Sirt1 inhibitor EX-527, and showed that EX-527 significantly blunted the stimulatory effect of flow on LC3 puncta accumulation (Figure 3h). EX-527 also suppressed flow-induced upregulation of beclin-1, Atg5, and LC3A (Figure 3i). Finally, we performed Sirt1 gene silencing experiments using siRNA as described before,^20^ and showed that knockdown of Sirt1 significantly inhibited the stimulatory effect of flow on LC3 puncta accumulation (Figure 3j).

### Flow-induced autophagy is redox dependent

**Figure 4:**
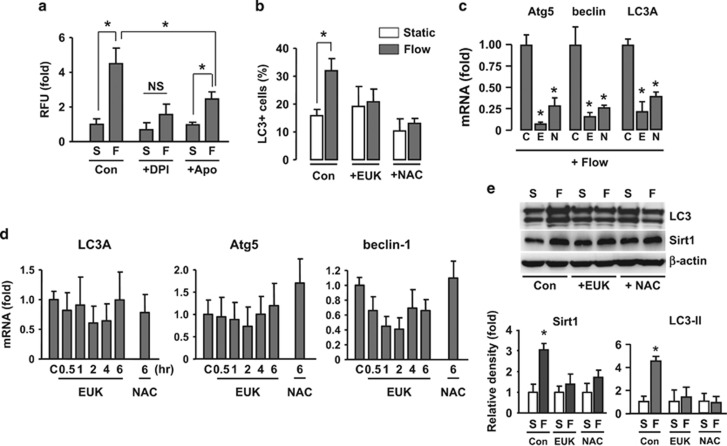
Shear stress-induced autophagy in EC was redox dependent. (**a**) ROS production measured with Amplex Red Hydrogen Peroxide Assay in cells maintained under laminar flow and static conditions, in the absence and presence of the NADPH oxidase inhibitors diphenyleneiodonium (DPI; 10 *μ*M) and diapocynin (100 *μ*M). (**b**) Effects of EUK-134 (10 *μ*M) and *N*-acetyl cysteine (NAC; 1 mM) on flow-induced LC3 puncta accumulation. (**c**) Effects of EUK-134 (E) and NAC (N) on the expression levels of Atg5, beclin-1, and LC3A in cells maintained under flow condition. (**d**) Effects of EUK-134 on the expression levels of Atg5, beclin-1, and LC3A in cells maintained under static condition. (**e**) Western blot and densitometry data showing the effects of laminar flow on protein levels of Sirt1 and LC3 in the absence and presence of EUK-134 or NAC. Data are mean±S.E.M. *P<0.05, unpaired *t*-test or one-way analysis of variance as appropriate (*n*=3–5). S, static; F, flow; NS, no significance; Apo, diapocynin; NAC, *N*-acetyl cysteine

It is well documented that shear stress regulates ROS production in EC, and redox-dependent mechanisms have important roles in mediating shear stress-induced responses,^21^ we next tested whether flow-induced autophagy was also related to redox regulation. We first measured the ROS production using the Amplex Red Hydrogen Peroxide Assay. We showed that laminar flow significantly increased the ROS production (Figure 4a). To confirm this result, we also performed DCFH-DA fluorescence. Similarly, we found that cells under flow condition displayed a higher intracellular ROS level than cells under static condition (Supplementary Figure VII). Numerous studies have demonstrated that NADPH oxidase is a major source of ROS in ECs.^22^ To confirm the source of ROS under current experimental settings, we treated the cells with various enzyme blockers. We showed that flow-induced ROS production was blocked by the NADPH oxidase inhibitors diphenyleneiodonium (DPI, 10 *μ*M) and diapocynin (100 *μ*M, purchased from Sigma; Figure 4a). In contrast, the nitric oxide synthase inhibitor *N*^G^-nitro-L-arginine methyl ester (100 *μ*M) or the mitochondrial respiration chain inhibitor rotenone (10 *μ*M) showed no effects on endothelial ROS generation (data not shown), which were consistent with our previous observations.^23^ Then we showed that pretreatment of the cells with EUK-134, a synthetic superoxide dismutase/catalase mimetic, significantly suppressed flow-induced autophagic response (Figure 4b). In cells maintained under flow condition, treatment with EUK-134 markedly decreased the expression levels of beclin-1, Atg5, and LC3A (Figure 4c), whereas EUK-134 had no significant effects in cells maintained under static condition (Figure 4d). Moreover, we showed that EUK-134 significantly blocked the upregulating effects of flow on Sirt1 and LC3-II (Figure 4e). To confirm the effects of EUK-134, we also pretreated the cells with another antioxidant *N*-acetyl cysteine (NAC, 1 mM). We showed that NAC produced similar inhibitory effects as EUK-134 on flow-induced autophagic responses (Figures 4b–e).

### Sirt1 is a redox-sensitive regulator of autophagy in ECs

**Figure 5:**
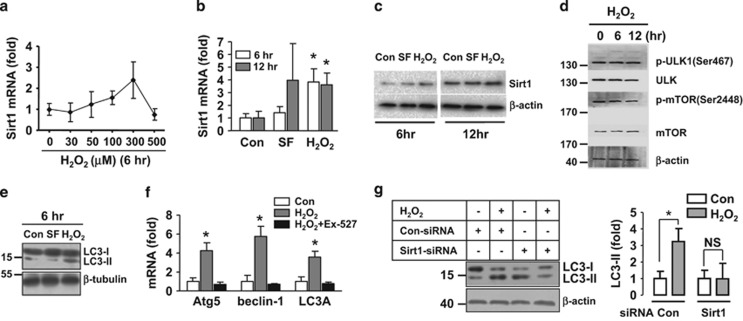
Sirt1 was a redox-sensitive regulator of reactive oxygen-induced autophagy. (**a**) Concentration-dependent effects of exogenous H_2_O_2_ on the expression level of Sirt1. (**b**) Effects of H_2_O_2_ (300 *μ*M) on Sirt1 mRNA expression at 6 and 12 h. Serum-free treatment was used as a control. (**c**) Effects of H_2_O_2_ and serum free on Sirt1 protein expression. (**d**) Effects of H_2_O_2_ on phosphorylation of mTOR and ULK1. (**e**) Effects of H_2_O_2_ and serum-free treatment on the protein level of LC3. (**f**) H_2_O_2_ increased the expression levels of Atg5, beclin-1, and LC3A, which were blocked by pretreatment with EX-527 (10 *μ*M). (**g**) Western blot and quantitative densitometry data showing the effects of control and Sirt1 siRNA on the autophagic response induced by H_2_O_2_ (300 *μ*M). Data are mean±S.E.M. *P<0.05, one-way analysis of variance, *n*=3–4. The western blots were representative examples from three experiments. NS, no significance; SF, serum free

Autophagy is regulated by intracellular redox status, whereas the mechanistic links between ROS and autophagy are poorly understood. Given the pivotal role of Sirt1 in maintaining cellular homeostasis during oxidative stress,^24^ we then examined whether Sirt1 could function as a redox-sensitive transducer. An oxidative condition was induced by treating cells with exogenous H_2_O_2_. Because the effects of H_2_O_2_ on Sirt1 expression appeared to be variable in the literature, we first performed a dose–response analysis on H_2_O_2_ effects. We found that at 300 *μ*M, H_2_O_2_ significantly increased the expression level of Sirt1 in EC (Figure 5a). Using this concentration, we further showed that H_2_O_2_ also increased both of the mRNA and protein levels of Sirt1 (Figures 5a and c). In contrast, H_2_O_2_ treatment had no effects on the phosphorylation levels of mTOR or ULK1 (Figure 5d). We next demonstrated that treatment with H_2_O_2_ increased EC autophagy as measured by LC3 western blot (Figure 5e). Moreover, we demonstrated that H_2_O_2_ increased the expression levels of beclin-1, Atg5, and LC3A, and these actions were all abrogated by inhibiting Sirt1 with EX-527 (Figure 5f). To further establish the role of Sirt1 in redox-dependent autophagy, we transfected the cells with Sirt1 siRNA and showed that H_2_O_2_-induced accumulation of LC3-II was abolished by Sirt1 siRNA (Figure 5g).

### Sirt1-dependent activation of FoxO1 is critical in mediating shear-induced autophagy

**Figure 6:**
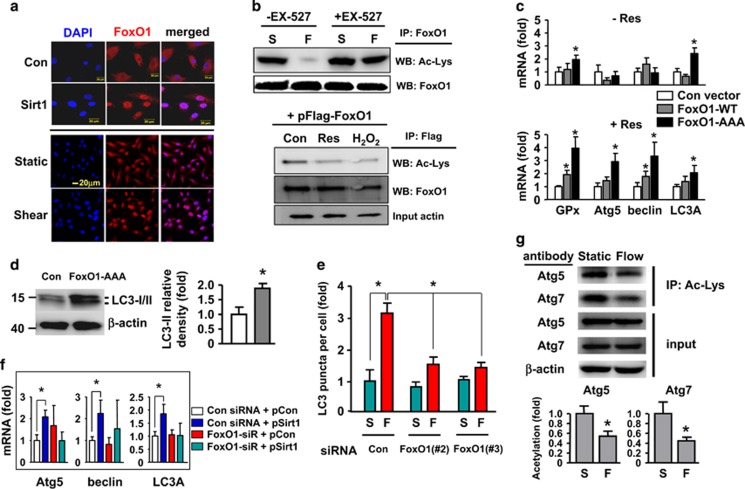
Role of FoxO1 in shear stress- and Sirt1-mediated autophagic response. (**a**) Immunofluorescence images showing the effects of laminar flow and Sirt1 overexpression on nuclear retention of FoxO1. Nuclei were counter-stained with DAPI (blue). (**b**) Effects of laminar flow, resveratrol (Res; 10 *μ*M), and H_2_O_2_ (300 *μ*M) on the acetylation level of FoxO1 in normal cells (upper) or cells expressing ectopic Flag-FoxO1 (lower). Cell lysates were immunoprecipitated with an anti-FoxO1 or anti-Flag antibody and detected by western blotting with an anti-acetyl lysine (Ac-Lys) antibody. (**c**) Effects of overexpressing wild type (WT) or the constitutively active form (AAA) of FoxO1 on the expression levels of glutathione peroxidase (GPx), Atg5, beclin-1, and LC3A, in the absence and presence of Res. (**d**) Overexpression of FoxO1-AAA enhanced the protein level of LC3-II in the presence of Res. (**e**) Effects of FoxO1 gene silencing with different siRNA sequences on laminar flow-induced autophagic response detected by LC3 immunofluorescence. (**f**) Effects of FoxO1 siRNA on Sirt1 overexpression (pSirt1)-induced upregulation of Atg5, beclin-1, and LC3A. (**g**) Laminar flow decreased the acetylation levels of Atg5 and Atg7 detected by immunoprecipitation (IP) with anti-acetyl lysine antibody and western blotting with anti-Atg5 and Atg7 antibodies. Data are mean±S.E.M. *P<0.05, one-way analysis of variance or unpaired *t*-test, *n*=4–6. S, static; F, flow; pCon, control plasmid; Res, resveratrol; IP, immunoprecipitation; WT, wild type; GPx, glutathione peroxidase

FoxO transcription factors are important downstream effectors of Sirt1. To clarify whether FoxO is involved in shear- and Sirt1-mediated autophagy in ECs, we first measured the responsiveness of FoxO1 to shear stress and Sirt1. As shown in Figure 6a, we found that both of flow and Sirt1 overexpression induced FoxO1 nuclear translocation. Treatment of the cells with resveratrol also produced similar effects (data not shown). Then we examined how shear stress and Sirt1 regulated FoxO1 acetylation. We showed that flow significantly decreased the acetylation level of FoxO1, indicating an increased interaction between Sirt1 and FoxO1 with shear stress stimulation; and this effect was inhibited by cotreatment with EX-527 (Figure 6b and Supplementary Figure VIII). Moreover, FoxO1 acetylation level was also reduced by resveratrol and H_2_O_2_ (Figure 6b and Supplementary Figure VIII).

Next, we overexpressed the wild-type FoxO1 and the constitutively active FoxO1-AAA mutant. Overexpression of both forms of FoxO1 increased the expression levels of beclin-1, Atg5, LC3A, and glutathione peroxidase-1 (a known target gene of FoxO1), while these effects were more prominent in the presence of resveratrol (Figure 6c). The little effects of wild-type FoxO1 in the absence of resveratrol may reflect the fact that FoxO1 activity is coordinately regulated by both phosphorylation and acetylation.^25^ Moreover, expression of FoxO1-AAA significantly increased the level of LC3-II and total LC3 in the presence of resveratrol (Figure 6d). Because FoxO3a is also expressed in EC and may have overlapping roles as FoxO1, we also tested the effects of FoxO3a. Nonetheless, we found that overexpression of FoxO3a produced non-significant effects in contrast to FoxO1, either in the absence or presence of resveratrol (data not shown). Then we tested effects of three different FoxO1 siRNAs; we found that siRNA #2 and #3 showed high gene silencing efficacies (Supplementary Figure IX). Using siRNA #2, we demonstrated that knocking down FoxO1 expression significantly inhibited the stimulatory effect of flow on autophagy (Figure 6e). To exclude possible off target actions of the siRNA, we repeated the experiments with siRNA #3, and confirmed that these siRNAs had similar effects (Figure 6e). Moreover, we demonstrated that FoxO1 gene silencing suppressed the effects of Sirt1 overexpression on the expression of autophagic genes beclin-1, Atg5, and LC3A (Figure 6f).

### Shear stress induces deacetylation of Atg5 and Atg7

There is evidence suggesting that Sirt1 may regulate autophagy through deacetylation of Atg proteins.^26^ Therefore, we performed immunoprecipitation and western blot experiments, and showed that treatment with flow indeed decreased the acetylation levels of Atg5 and Atg7 (Figure 6g).

### Flow-induced autophagy was cytoprotective in EC

**Figure 7:**
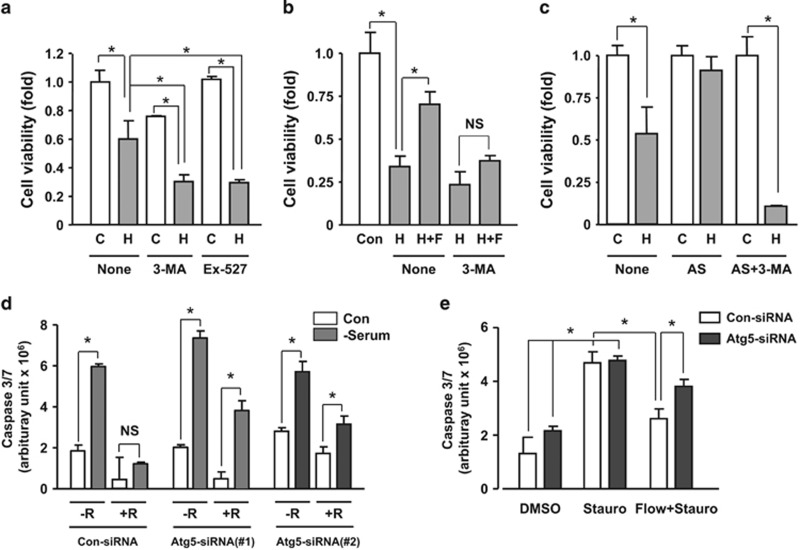
Autophagic response had cytoprotective actions in endothelial cells. (**a**) Flow-adapted cells were pretreated with the autophagy inhibitor 3-MA (10 *μ*M) or the Sirt1 inhibitor EX-527, and acutely challenged with a high concentration (600 *μ*M) of H_2_O_2_ for 2 h. (**b**) Effects of laminar flow adaptation on H_2_O_2_-induced cell death in the absence and presence of 3-MA. (**c**) Effects of autophagy induction by amino-acid starvation (by incubating cells in Hank's balanced salt solution for 4 h) in static cells on H_2_O_2_-induced cell death. (**d**) Effects of Atg5 gene silencing with different siRNA sequences on the cytoprotective effects of resveratrol (10 *μ*M) assessed by serum deprivation-induced cell apoptosis. (**e**) Effects of Atg5 gene silencing on the cytoprotective effects of laminar flow assessed by staurosporine (100 nM)-induced cell apoptosis. Data are mean±S.E.M. *P<0.05, one-way analysis of variance, *n*=4–5. H, H_2_O_2_; F, flow; AS, amino-acid starvation; R, resveratrol; Stauro, staurosporine; C, control; NS, no significance

To understand the functional importance of autophagy in EC, we pretreated flow-adapted cells with the autophagy inhibitor 3-methyladenine (3-MA) and the Sirt1 inhibitor EX-527, and induced acute cellular injuries using a high concentration of H_2_O_2_ (600 *μ*M for 2 h). We showed that 3-MA and EX-527 aggravated oxidant-induced cell death (Figure 7a). Next, we compared the H_2_O_2_-induced cell death in static-adapted and flow-adapted (20 dyn/cm^2^ for 8 h) cells. We found that flow significantly decreased H_2_O_2_-induced cell death, an effect that was diminished by 3-MA cotreatment (Figure 7b). To confirm that the cytoprotective effect of flow observed in Figure 7b was associated with changes in the level of autophagy, we detected LC3 immunofluorescence under these treatment conditions. We showed that the short-term ROS challenge did not trigger autophagic responses, whereas flow-adapted cells showed increased autophagy. Moreover, flow-induced autophagy was blocked by 3-MA (Supplementary Figure X). To clarify whether induction of autophagy *per se* in the absence of shear stress was also cytoprotective, we induced autophagy by amino-acid starvation and demonstrated that autophagy induction suppressed H_2_O_2_-induced cell death, which was abolished by 3-MA (Figure 7c). Moreover, we knocked down Atg5 expression with two different siRNA sequences (Supplementary Figure XI). We showed that Atg5 siRNAs partially attenuated the cytoprotective effect of resveratrol on cell apoptosis induced by serum deprivation (Figure 7d). In addition, as shown in Figure 7e, we confirmed that Atg5 gene silencing also partially reversed the cytoprotective effect of shear stress using staurosporine as an apoptosis inducer. These results were consistent with previous studies showing that autophagy protected cells from both necrotic ^27, 28^ and apoptotic cell death.^29, 30, 31^

## Discussion

Here we have provided evidence suggesting that shear stress induced by laminar flow can promote autophagy in vascular ECs. This response was unlikely to be due to reduced autophagic flux, as evidenced by the similar effect of flow in the presence of bafilomycin and the unchanged p62 level. Studies using loss-of-function models have suggested that a normal autophagic process is important for maintaining cellular homeostasis in myocardium, skeletal muscle, and neurons.^32, 33, 34^ In vascular EC, disruption of autophagy by genetic manipulation of beclin-1 or LC3B had no effect on EC phenotype under normal conditions; but autophagy-deficient ECs displayed aberrant cellular functions under various stress conditions.^35, 36^ In addition, we found that flow-conditioned cells are more resistant to oxidant-induced cell death, and this beneficial effect was abolished after pharmacological and genetic inhibition of autophagy. This observation is consistent with previous studies showing that under certain circumstances, inhibition of the autophagic process in EC increased the cell sensitivity to various stress stimuli,^6, 10, 29, 37^ whereas induction of autophagy in EC had cytoprotective actions.^30, 31, 38^ Given the well-documented protective effects of laminar blood flow on normal endothelial functions, taken together, our results suggest that induction of autophagy may represent a novel mechanism of the vascular-protective actions of laminar flow.

Mammalian Sirt1 has a pivotal role in modulating stress responses in mammalian cells.^39^ Emerging evidence has suggested that Sirt1 may have an important role in modulating cell autophagy.^26, 40, 41, 42^ In cultured cardiac myocytes, nutrient deprivation upregulated expression of Sirt1 and induced autophagy, which was attenuated by Sirt1 inhibition.^40^ Moreover, in kidney proximal tubular cells, inhibiting Sirt1 function significantly inhibited the autophagic responses induced by calorie restriction.^41^ Our observation that flow upregulated Sirt1 expression in EC was consistent with those reported by others.^43, 44^ Using both gain- and loss-of-function experiments, we provided evidence supporting that Sirt1-mediated FoxO1 activation was likely to have a major role in flow-induced autophagy in vascular EC. In addition, we found that flow-induced Sirt1 upregulation and autophagy were accompanied by reductions in the acetylation level of Atg5 and Atg7, an observation that was consistent with that obtained in HeLa cells.^26^ Hence, we could not exclude that Sirt1-mediated autophagy in EC might involve multiple mechanisms.

We showed that flow increased ROS production in EC, which was consistent with previous results.^45, 46, 47, 48, 49^ In addition, antioxidant treatment prevented flow-induced autophagy, and exogenous H_2_O_2_ mimicked the effects of flow, consistent with previous studies in neurons.^50^ It is clear that autophagy is regulated by intracellular redox status;^6, 51^ however, the mechanistic links between ROS and autophagy are still poorly understood.^52, 53^ Our data have shed a light on this question. We found that an oxidative condition enhanced Sirt1 expression, whereas blockade of the Sirt1 function suppressed ROS-induced autophagic responses. The regulatory effects of ROS on Sirt1 expression appear to be divergent. Both of stimulatory and inhibitory effects of ROS on Sirt1 at the transcriptional level have been reported in different cells.^54, 55, 56^ Moreover, ROS may also regulate Sirt1 expression at the posttranscriptional and posttranslational levels.^57, 58^ It appears that the final outcome is in a highly cell- and context-dependent manner. Our observation is supported by several studies showing that ROS may upregulate the expression of Sirt1.^54, 55, 59, 60^ The molecular mechanisms of ROS-dependent regulation of Sirt1 expression are not entirely understood. There is evidence showing that H_2_O_2_ treatment increases the Sirt1 promoter activity, indicating an important role of redox-dependent regulation of the Sirt1 gene transcription.^61^ Likewise, we observed that shear stress enhanced the Sirt1 promoter activity in EC. We suggest that Sirt1 may act as an intracellular redox sensor that mediates ROS-triggered autophagy response in EC.

Functions of FoxO are enhanced by deacetylation.^25^ We demonstrated that flow decreased the acetylation level of FoxO1 in EC; likewise, we showed that overexpression of Sirt1 induced nuclear localization of FoxO1 in EC. FoxO regulates the expression of multiple autophagy-related genes, including LC3, Atg5, Atg8, Atg12, Bnip3, and beclin-1.^41, 42, 62^ Moreover, there is evidence showing that FoxO can modulate the transcription of some autophagic genes by directly binding to the promoter region.^41, 42^ Our data suggest that flow may enhance FoxO functions in EC via upregulation of Sirt1, whereas FoxO activation in turn promotes the autophagic response. Indeed, important roles of the Sirt1-FoxO axis in modulating autophagy have been documented in previous studies in cardiomyocytes and skeletal muscle cells,^40, 42, 63^ although the results appear controversial. It is noted that the mechanisms by which FoxO modulates autophagy appear to be complex,^40, 64^ whereas transcriptional regulation of autophagic genes may not be able to fully explain the effect of FoxO. Therefore, more studies are needed to further elucidate the mechanisms by which the Sirt1/FoxO pathway regulates autophagy in EC.

In summary, we have presented evidence showing that laminar flow-induced shear stress promotes autophagic responses in ECs via a redox- and Sirt1-dependent mechanism. Shear stress-induced autophagy in ECs may represent a novel mechanism by which laminar blood flow produces its vascular-protective actions.

## Materials and Methods

### Reagents

Resveratrol, EX-527, and 3-MA were purchased from Sigma (St. Louis, MO, USA). Euk-134 was from Cayman Chemicals (Ann Arbor, MI, USA). Plasmids expressing wild-type Sirt1 (pFlag-Sirt1) and the deacetylase-dead mutant (pFlag-Sirt1-H363Y),^39^ wild-type FoxO1 (pFlag-FoxO1) and the constitutively active form of FoxO1 (pFlag- FoxO1-AAA)^65^ were obtained from Addgene (Cambridge, MA, USA). The plasmid expressing GFP-tagged LC3 (pSelect-GFP-LC3) was purchased from InvivoGen (San Diego, CA, USA). Sequences of siRNA targeting human Sirt1 and FoxO1 were synthesized by GenePharma (Shanghai, China). A non-targeting siRNA (5′-UUCUCCGAACGUGUCACGUTT-3′) was used as control.

### Cell culture

HUVECs and telomerase-immortalized human microvascular endothelium cell line (TIME cells) were purchased from the American Type Culture Collection (Rockville, MD, USA). Cells were maintained in complete ECM medium (Catalog #1001, ScienCell, Carlsbad, CA, USA) supplemented with 5% FBS, the Endothelial Cell Growth Supplement, penicillin (100 U/ml), and streptomycin (100 *μ*g/ml) as described.^66^

### *In vitro* flow simulation

Cells were seeded on glass slides coated with collagen and cultured in a Streamer parallel-plate flow chamber (FlexCell, Burlington, NC, USA) system. The flow rate and type were controlled by a programmable flow controller device (Osci-Flow, FlexCell).

### Immunofluorescence and confocal microscopy

Cells cultured on Lab-Tek II chamber slides were fixed in 4% paraformaldehyde, permeabilized with 0.5% Triton X-100 for 15 min, and blocked with 2% BSA for 30 min. Cells were then incubated with anti-LC3 antibody (Cell Signaling, Beverley, MA, USA) overnight followed by FITC-conjugated anti-IgG (Jackson ImmunoResearch Laboratories, West Grove, PA, USA) at room temperature for 1 h. DAPI was used for counter-staining. Images were taken with a laser-scanning confocal microscope (Model LSM710, Zeiss, Jena, Germany). LC3 morphology was assessed by an independent viewer in a blind manner. For each independent experiment, 5–10 random high-power fields (at least 50 cells in total) were surveyed. Cells were arbitrarily categorized into punctate LC3+ and LC3- groups (as guidance, LC3 punctation induced by amino-acid starvation was used as a positive control). We also counted the average number of LC3 puncta in individual cells.

### Western blot and immunoprecipitation

Cells were homogenized in cold lysis buffer (50 mM Tris, pH 7.5, 2 mM EDTA, 100 mM NaCl, 50 mM NaF, 1% Triton X-100, 1 mM Na_3_VO_4_, and 40 mM *β*-glycerol phosphate) containing a protease inhibitor cocktail (Roche, Mannheim, Germany). Total proteins were separated by SDS-PAGE and electro-blotted onto nitrocellulose or PVDF membranes. After blocking with 5% non-fat milk, membranes were probed using various primary antibodies at 4 °C for overnight, followed by 2 h of incubation with horseradish peroxidase-conjugated secondary antibodies at room temperature. The membranes were developed with an enhanced chemiluminescence reagent (Millipore, Temecula, CA, USA). The following antibodies were used: Sirt1, LC3B, phospho- and total ULK1, phospho- and total mTOR, Flag tag, acetylated-lysine (all from Cell Signaling), and FoxO1A (from Abcam, Cambridge, UK). For immunoprecipitation, cell lysates were precleared and incubated with 2 *μ*g of capture antibody and 20 *μ*l of 50% protein A/G-agarose bead slurry (Pierce Biotechnology, Rockford, IL, USA) overnight at 4 °C with gentle rotation. The beads were washed and boiled in 2 × Laemmli buffer. The densitometry analysis was performed with Image-J software (NIH, Bethesda, MD, USA). The specificity of antibodies used for immunoprecipitation was routinely validated by running negative controls using non-immune IgG using the same conditions as in formal experiments.

### Quantitative real-time PCR

Total RNA was isolated with TRIzol reagent (Life Technologies, Carlsbad, CA, USA) and reverse transcribed using random hexamers and the PrimeScript RT Kit from TaKaRa (Dalian, China). Quantitative real-time PCR was performed using predesigned Taqman probe-primer sets and the Gene Expression Master Mix in a Prism 7500 system (all from Applied Biosystems, Foster City, CA, USA). 18 S was used as the house keeping gene. The 2^−ΔΔCt^ method was used to assess the relative mRNA expression level.

### Sirt1 promoter reporter assay

The human Sirt1 promoter sequence (−1616 to +5 bp) with 5' *Sac*I and 3' *Hin*dIII sites was obtained by DNA synthesis (BioSune, Shanghai, China) and cloned into pGL3 vector. Cells were transfected with Lipofectamine LTX Reagent (Life Technologies), and the reporter activity was measured with a Luciferase Assay Kit (Promega, Madison, WI, USA).

### Intracellular ROS measurement

ROS production was measured with an Amplex Red Hydrogen Peroxide Assay Kit (Life Technologies) as described in our previous studies.^67^ In addition, DCFH-DA (Life Technologies) fluorescence was also used to measure intracellular ROS levels. Briefly, cells were incubated with 5 *μ*M DCFH-DA at 37 °C for 20 min in Hanks' balanced salt solution. After staining, cells were rinsed and photographed with a fluorescent microscope (Nikon Eclipse 80i, Nikon, Melville, NY, USA). The fluorescent intensity was measured with Image-Pro Plus software (Media Cybernetics, Atlanta, GA, USA).

### Plasmid and siRNA transfection

Cells were subcultured into 24-well plates 24 h before transfection and maintained in antibiotic-free Opti-MEM medium (Life Technologies). Plasmid transfection was performed using 500 ng DNA and the Lipofectamine 2000 reagent (Life Technologies). Transfection of siRNA was performed at 30 nM of final concentration using the Lipofectamine RNAiMAX reagent (Life Technologies). After 6 h of treatment, the cells were changed to fresh complete culture medium.

### Cell viability assays

Cell viability was assessed with the tetrazolium-based (MTS) assay using CellTiter 96 Aqueous kit (from Promega) according to the manufacturer's direction. Apoptosis was assessed by a Caspase-Glo caspase3/7 activity assay kit (Promega).

### Statistical analysis

Data are presented as mean±standard error of the mean (S.E.M.). Data analysis was performed with unpaired *t*-test or one-way analysis of variance followed by *post-hoc* Tukey's test as appropriate. A *P* value of <0.05 was considered as statistically significant.

## Abbreviations

EC: endothelial cell
mTOR: mammalian target of rapamycin
ROS: reactive oxygen species
HUVEC: human umbilical vein endothelial cell
TIME: hTERT-immortalized human dermal microvascular endothelial cell line
DPI: diphenyleneiodonium
NAC: *N*-acetyl cysteine
3-MA: 3-methyladenine

## References

[bib1] 1Davies PF. Hemodynamic shear stress and the endothelium in cardiovascular pathophysiology. Nat Clin Pract Cardiovasc Med 2009; 6: 16–26. 19029993 10.1038/ncpcardio1397 PMC2851404

[bib2] 2Chiu JJ, Chien S. Effects of disturbed flow on vascular endothelium: pathophysiological basis and clinical perspectives. Physiol Rev 2011; 91: 327–387. 21248169 10.1152/physrev.00047.2009 PMC3844671

[bib3] 3Mizushima N. Autophagy: process and function. Genes Dev 2007; 21: 2861–2873. 18006683 10.1101/gad.1599207

[bib4] 4Ravikumar B, Sarkar S, Davies JE, Futter M, Garcia-Arencibia M, Green-Thompson ZW et al. Regulation of mammalian autophagy in physiology and pathophysiology. Physiol Rev 2010; 90: 1383–1435. 20959619 10.1152/physrev.00030.2009

[bib5] 5Glick D, Barth S, Macleod KF. Autophagy: cellular and molecular mechanisms. J Pathol 2010; 221: 3–12. 20225336 10.1002/path.2697 PMC2990190

[bib6] 6Wang Q, Liang B, Shirwany NA, Zou MH. 2-Deoxy-D-glucose treatment of endothelial cells induces autophagy by reactive oxygen species-mediated activation of the AMP-activated protein kinase. PLoS One 2011; 6: e17234. 21386904 10.1371/journal.pone.0017234 PMC3046135

[bib7] 7Csordas A, Kreutmayer S, Ploner C, Braun PR, Karlas A, Backovic A et al. Cigarette smoke extract induces prolonged endoplasmic reticulum stress and autophagic cell death in human umbilical vein endothelial cells. Cardiovasc Res 2011; 92: 141–148. 21676957 10.1093/cvr/cvr165

[bib8] 8Bharath LP, Mueller R, Li Y, Ruan T, Kunz D, Goodrich R et al. Impairment of autophagy in endothelial cells prevents shear-stress-induced increases in nitric oxide bioavailability. Can J Physiol Pharmacol 2014; 92: 605–612. 24941409 10.1139/cjpp-2014-0017 PMC8370712

[bib9] 9Khan MJ, Rizwan Alam M, Waldeck-Weiermair M, Karsten F, Groschner L, Riederer M et al. Inhibition of autophagy rescues palmitic acid-induced necroptosis of endothelial cells. J Biol Chem 2012; 287: 21110–21120. 22556413 10.1074/jbc.M111.319129 PMC3375534

[bib10] 10Higdon AN, Benavides GA, Chacko BK, Ouyang X, Johnson MS, Landar A et al. Hemin causes mitochondrial dysfunction in endothelial cells through promoting lipid peroxidation: the protective role of autophagy. Am J Physiol Heart Circ Physiol 2012; 302: H1394–H1409. 22245770 10.1152/ajpheart.00584.2011 PMC3330785

[bib11] 11Du J, Teng RJ, Guan T, Eis A, Kaul S, Konduri GG et al. Role of autophagy in angiogenesis in aortic endothelial cells. Am J Physiol Cell Physiol 2012; 302: C383–C391. 22031599 10.1152/ajpcell.00164.2011 PMC3328843

[bib12] 12Patschan S, Chen J, Polotskaia A, Mendelev N, Cheng J, Patschan D et al. Lipid mediators of autophagy in stress-induced premature senescence of endothelial cells. Am J Physiol Heart Circ Physiol 2008; 294: H1119–H1129. 18203850 10.1152/ajpheart.00713.2007

[bib13] 13Guo F, Li X, Peng J, Tang Y, Yang Q, Liu L et al. Autophagy regulates vascular endothelial cell eNOS and ET-1 expression induced by laminar shear stress in an ex vivo perfused system. Ann Biomed Eng 2014; 42: 1978–1988. 24838486 10.1007/s10439-014-1033-5

[bib14] 14Takizawa Y, Kosuge Y, Awaji H, Tamura E, Takai A, Yanai T et al. Up-regulation of endothelial nitric oxide synthase (eNOS), silent mating type information regulation 2 homologue 1 (SIRT1) and autophagy-related genes by repeated treatments with resveratrol in human umbilical vein endothelial cells. Br J Nutr 2013; 110: 2150–2155. 23750556 10.1017/S0007114513001670

[bib15] 15Filomeni G, De Zio D, Cecconi F. Oxidative stress and autophagy: the clash between damage and metabolic needs. Cell Death Differ 2015; 22: 377–388. 25257172 10.1038/cdd.2014.150 PMC4326572

[bib16] 16Urbanek T, Kuczmik W, Basta-Kaim A, Gabryel B. Rapamycin induces of protective autophagy in vascular endothelial cells exposed to oxygen-glucose deprivation. Brain Res 2014; 1553: 1–11. 24462935 10.1016/j.brainres.2014.01.017

[bib17] 17Yang C, Kaushal V, Shah SV, Kaushal GP. Autophagy is associated with apoptosis in cisplatin injury to renal tubular epithelial cells. Am J Physiol Renal Physiol 2008; 294: F777–F787. 18256309 10.1152/ajprenal.00590.2007

[bib18] 18Son YO, Pratheeshkumar P, Roy RV, Hitron JA, Wang L, Zhang Z et al. Nrf2/p62 signaling in apoptosis resistance and its role in cadmium-induced carcinogenesis. J Biol Chem 2014; 289: 28660–28675. 25157103 10.1074/jbc.M114.595496 PMC4192515

[bib19] 19Wang L, Cano M, Handa JT. p62 provides dual cytoprotection against oxidative stress in the retinal pigment epithelium. Biochim Biophys Acta 2014; 1843: 1248–1258. 24667411 10.1016/j.bbamcr.2014.03.016 PMC4019388

[bib20] 20Liu J, Wu X, Wang X, Zhang Y, Bu P, Zhang Q et al. Global gene expression profiling reveals functional importance of Sirt2 in endothelial cells under oxidative stress. Int J Mol Sci 2013; 14: 5633–5649. 23478437 10.3390/ijms14035633 PMC3634502

[bib21] 21Cunningham KS, Gotlieb AI. The role of shear stress in the pathogenesis of atherosclerosis. Lab Invest 2005; 85: 9–23. 15568038 10.1038/labinvest.3700215

[bib22] 22Frey RS, Ushio-Fukai M, Malik AB. NADPH oxidase-dependent signaling in endothelial cells: role in physiology and pathophysiology. Antioxid Redox Signal 2009; 11: 791–810. 18783313 10.1089/ars.2008.2220 PMC2790033

[bib23] 23Jiang F, Roberts SJ, Datla Sr, Dusting GJ. NO modulates NADPH oxidase function via heme oxygenase-1 in human endothelial cells. Hypertension 2006; 48: 950–957. 16982957 10.1161/01.HYP.0000242336.58387.1f

[bib24] 24Webster BR, Lu Z, Sack MN, Scott I. The role of sirtuins in modulating redox stressors. Free Radic Biol Med 2012; 52: 281–290. 22085655 10.1016/j.freeradbiomed.2011.10.484 PMC3253188

[bib25] 25Qiang L, Banks AS, Accili D. Uncoupling of acetylation from phosphorylation regulates FoxO1 function independent of its subcellular localization. J Biol Chem 2010; 285: 27396–27401. 20519497 10.1074/jbc.M110.140228 PMC2930737

[bib26] 26Lee IH, Cao L, Mostoslavsky R, Lombard DB, Liu J, Bruns NE et al. A role for the NAD-dependent deacetylase Sirt1 in the regulation of autophagy. Proc Natl Acad Sci U S A 2008; 105: 3374–3379. 18296641 10.1073/pnas.0712145105 PMC2265142

[bib27] 27Shen HM, Codogno P. Autophagy is a survival force via suppression of necrotic cell death. Exp Cell Res 2012; 318: 1304–1308. 22366289 10.1016/j.yexcr.2012.02.006

[bib28] 28Ryter SW, Mizumura K, Choi AM. The impact of autophagy on cell death modalities. Int J Cell Biol 2014; 2014: 502676. 24639873 10.1155/2014/502676 PMC3932252

[bib29] 29Nishikawa T, Tsuno NH, Okaji Y, Sunami E, Shuno Y, Sasaki K et al. The inhibition of autophagy potentiates anti-angiogenic effects of sulforaphane by inducing apoptosis. Angiogenesis 2010; 13: 227–238. 20694744 10.1007/s10456-010-9180-2

[bib30] 30Dong Z, Wang L, Xu J, Li Y, Zhang Y, Zhang S et al. Promotion of autophagy and inhibition of apoptosis by low concentrations of cadmium in vascular endothelial cells. Toxicol In Vitro 2009; 23: 105–110. 19061949 10.1016/j.tiv.2008.11.003

[bib31] 31Wei DH, Jia XY, Liu YH, Guo FX, Tang ZH, Li XH et al. Cathepsin L stimulates autophagy and inhibits apoptosis of ox-LDL-induced endothelial cells: potential role in atherosclerosis. Int J Mol Med 2013; 31: 400–406. 23229094 10.3892/ijmm.2012.1201

[bib32] 32Nakai A, Yamaguchi O, Takeda T, Higuchi Y, Hikoso S, Taniike M et al. The role of autophagy in cardiomyocytes in the basal state and in response to hemodynamic stress. Nat Med 2007; 13: 619–624. 17450150 10.1038/nm1574

[bib33] 33Wu JJ, Quijano C, Chen E, Liu H, Cao L, Fergusson MM et al. Mitochondrial dysfunction and oxidative stress mediate the physiological impairment induced by the disruption of autophagy. Aging (Albany NY) 2009; 1: 425–437. 20157526 10.18632/aging.100038 PMC2806022

[bib34] 34Hara T, Nakamura K, Matsui M, Yamamoto A, Nakahara Y, Suzuki-Migishima R et al. Suppression of basal autophagy in neural cells causes neurodegenerative disease in mice. Nature 2006; 441: 885–889. 16625204 10.1038/nature04724

[bib35] 35Lee SJ, Kim HP, Jin Y, Choi AM, Ryter SW. Beclin 1 deficiency is associated with increased hypoxia-induced angiogenesis. Autophagy 2011; 7: 829–839. 21685724 10.4161/auto.7.8.15598 PMC3149693

[bib36] 36Lee SJ, Smith A, Guo L, Alastalo TP, Li M, Sawada H et al. Autophagic protein LC3B confers resistance against hypoxia-induced pulmonary hypertension. Am J Respir Crit Care Med 2011; 183: 649–658. 20889906 10.1164/rccm.201005-0746OC PMC3081281

[bib37] 37Chen G, Zhang W, Li YP, Ren JG, Xu N, Liu H et al. Hypoxia-induced autophagy in endothelial cells: a double-edged sword in the progression of infantile haemangioma? Cardiovasc Res 2013; 98: 437–448. 23408345 10.1093/cvr/cvt035

[bib38] 38Han J, Pan XY, Xu Y, Xiao Y, An Y, Tie L et al. Curcumin induces autophagy to protect vascular endothelial cell survival from oxidative stress damage. Autophagy 2012; 8: 812–825. 22622204 10.4161/auto.19471

[bib39] 39Brunet A, Sweeney LB, Sturgill JF, Chua KF, Greer PL, Lin Y et al. Stress-dependent regulation of FOXO transcription factors by the SIRT1 deacetylase. Science 2004; 303: 2011–2015. 14976264 10.1126/science.1094637

[bib40] 40Hariharan N, Maejima Y, Nakae J, Paik J, Depinho RA, Sadoshima J. Deacetylation of FoxO by Sirt1 Plays an Essential Role in Mediating Starvation-Induced Autophagy in Cardiac Myocytes. Circ Res 2010; 107: 1470–1482. 20947830 10.1161/CIRCRESAHA.110.227371 PMC3011986

[bib41] 41Kume S, Uzu T, Horiike K, Chin-Kanasaki M, Isshiki K, Araki S et al. Calorie restriction enhances cell adaptation to hypoxia through Sirt1-dependent mitochondrial autophagy in mouse aged kidney. J Clin Invest 2010; 120: 1043–1055. 20335657 10.1172/JCI41376 PMC2846062

[bib42] 42Sengupta A, Molkentin JD, Yutzey KE. FoxO transcription factors promote autophagy in cardiomyocytes. J Biol Chem 2009; 284: 28319–28331. 19696026 10.1074/jbc.M109.024406 PMC2788882

[bib43] 43Chen Z, Peng IC, Cui X, Li YS, Chien S, Shyy JY. Shear stress, SIRT1, and vascular homeostasis. Proc Natl Acad Sci USA 2010; 107: 10268–10273. 20479254 10.1073/pnas.1003833107 PMC2890429

[bib44] 44Yao QP, Qi YX, Zhang P, Cheng BB, Yan ZQ, Jiang ZL. SIRT1 and Connexin40 Mediate the normal shear stress-induced inhibition of the proliferation of endothelial cells co-cultured with vascular smooth muscle cells. Cell Physiol Biochem 2013; 31: 389–399. 23548481 10.1159/000343376

[bib45] 45Hsieh HJ, Cheng CC, Wu ST, Chiu JJ, Wung BS, Wang DL. Increase of reactive oxygen species (ROS) in endothelial cells by shear flow and involvement of ROS in shear-induced c-fos expression. J Cell Physiol 1998; 175: 156–162. 9525474 10.1002/(SICI)1097-4652(199805)175:2<156::AID-JCP5>3.0.CO;2-N

[bib46] 46Chiu JJ, Wung BS, Shyy JY, Hsieh HJ, Wang DL. Reactive oxygen species are involved in shear stress-induced intercellular adhesion molecule-1 expression in endothelial cells. Arterioscler Thromb Vasc Biol 1997; 17: 3570–3577. 9437207 10.1161/01.atv.17.12.3570

[bib47] 47Breton-Romero R, Gonzalez de Orduna C, Romero N, Sanchez-Gomez FJ, de Alvaro C, Porras A et al. Critical role of hydrogen peroxide signaling in the sequential activation of p38 MAPK and eNOS in laminar shear stress. Free Radic Biol Med 2012; 52: 1093–1100. 22281399 10.1016/j.freeradbiomed.2011.12.026

[bib48] 48Han Z, Varadharaj S, Giedt RJ, Zweier JL, Szeto HH, Alevriadou BR. Mitochondria-derived reactive oxygen species mediate heme oxygenase-1 expression in sheared endothelial cells. J Pharmacol Exp Ther 2009; 329: 94–101. 19131585 10.1124/jpet.108.145557 PMC2670602

[bib49] 49Warabi E, Takabe W, Minami T, Inoue K, Itoh K, Yamamoto M et al. Shear stress stabilizes NF-E2-related factor 2 and induces antioxidant genes in endothelial cells: role of reactive oxygen/nitrogen species. Free Radic Biol Med 2007; 42: 260–269. 17189831 10.1016/j.freeradbiomed.2006.10.043

[bib50] 50Underwood BR, Imarisio S, Fleming A, Rose C, Krishna G, Heard P et al. Antioxidants can inhibit basal autophagy and enhance neurodegeneration in models of polyglutamine disease. Hum Mol Genet 2010; 19: 3413–3429. 20566712 10.1093/hmg/ddq253 PMC2916709

[bib51] 51Shen W, Tian C, Chen H, Yang Y, Zhu D, Gao P et al. Oxidative stress mediates chemerin-induced autophagy in endothelial cells. Free Radic Biol Med 2013; 55: 73–82. 23195684 10.1016/j.freeradbiomed.2012.11.011

[bib52] 52Huang J, Lam GY, Brumell JH. Autophagy signaling through reactive oxygen species. Antioxid Redox Signal 2011; 14: 2215–2231. 20874258 10.1089/ars.2010.3554

[bib53] 53Lee J, Giordano S, Zhang J. Autophagy mitochondria and oxidative stress: cross-talk and redox signalling. Biochem J 2012; 441: 523–540. 22187934 10.1042/BJ20111451 PMC3258656

[bib54] 54Prozorovski T, Schulze-Topphoff U, Glumm R, Baumgart J, Schroter F, Ninnemann O et al. Sirt1 contributes critically to the redox-dependent fate of neural progenitors. Nat Cell Biol 2008; 10: 385–394. 18344989 10.1038/ncb1700

[bib55] 55Brandl A, Meyer M, Bechmann V, Nerlich M, Angele P. Oxidative stress induces senescence in human mesenchymal stem cells. Exp Cell Res 2011; 317: 1541–1547. 21376036 10.1016/j.yexcr.2011.02.015

[bib56] 56Peng CH, Chang YL, Kao CL, Tseng LM, Wu CC, Chen YC et al. SirT1—a sensor for monitoring self-renewal and aging process in retinal stem cells. Sensors (Basel) 2010; 10: 6172–6194. 22219708 10.3390/s100606172 PMC3247753

[bib57] 57Abdelmohsen K, Pullmann R Jr., Lal A, Kim HH, Galban S, Yang X et al. Phosphorylation of HuR by Chk2 regulates SIRT1 expression. Mol Cell 2007; 25: 543–557. 17317627 10.1016/j.molcel.2007.01.011 PMC1986740

[bib58] 58Caito S, Rajendrasozhan S, Cook S, Chung S, Yao H, Friedman AE et al. SIRT1 is a redox-sensitive deacetylase that is post-translationally modified by oxidants and carbonyl stress. FASEB J 2010; 24: 3145–3159. 20385619 10.1096/fj.09-151308 PMC2923349

[bib59] 59Brandl A, Hartmann A, Bechmann V, Graf B, Nerlich M, Angele P. Oxidative stress induces senescence in chondrocytes. J Orthop Res 2011; 29: 1114–1120. 21284033 10.1002/jor.21348

[bib60] 60Hasegawa K, Wakino S, Yoshioka K, Tatematsu S, Hara Y, Minakuchi H et al. Sirt1 protects against oxidative stress-induced renal tubular cell apoptosis by the bidirectional regulation of catalase expression. Biochem Biophys Res Commun 2008; 372: 51–56. 18485895 10.1016/j.bbrc.2008.04.176

[bib61] 61Antoniali G, Lirussi L, D'Ambrosio C, Dal Piaz F, Vascotto C, Casarano E et al. SIRT1 gene expression upon genotoxic damage is regulated by APE1 through nCaRE-promoter elements. Mol Biol Cell 2014; 25: 532–547. 24356447 10.1091/mbc.E13-05-0286 PMC3923644

[bib62] 62Xu P, Das M, Reilly J, Davis RJ. JNK regulates FoxO-dependent autophagy in neurons. Genes Dev 2011; 25: 310–322. 21325132 10.1101/gad.1984311 PMC3042155

[bib63] 63Lee D, Goldberg AL. SIRT1 protein, by blocking the activities of transcription factors FoxO1 and FoxO3, inhibits muscle atrophy and promotes muscle growth. J Biol Chem 2013; 288: 30515–30526. 24003218 10.1074/jbc.M113.489716 PMC3798522

[bib64] 64Zhao Y, Yang J, Liao W, Liu X, Zhang H, Wang S et al. Cytosolic FoxO1 is essential for the induction of autophagy and tumour suppressor activity. Nat Cell Biol 2010; 12: 665–675. 20543840 10.1038/ncb2069

[bib65] 65Tang ED, Nunez G, Barr FG, Guan KL. Negative regulation of the forkhead transcription factor FKHR by Akt. J Biol Chem 1999; 274: 16741–16746. 10358014 10.1074/jbc.274.24.16741

[bib66] 66Cheng HS, Sivachandran N, Lau A, Boudreau E, Zhao JL, Baltimore D et al. MicroRNA-146 represses endothelial activation by inhibiting pro-inflammatory pathways. EMBO Mol Med 2013; 5: 949–966. 10.1002/emmm.201202318 PMC3721471 23733368

[bib67] 67Peshavariya HM, Chan EC, Liu GS, Jiang F, Dusting GJ. Transforming growth factor-beta1 requires NADPH oxidase 4 for angiogenesis *in vitro* and *in vivo*. J Cell Mol Med 2014; 18: 1172–1183. 24629065 10.1111/jcmm.12263 PMC4508156

