# Supplementary material for: Shear stress regulates endothelial cell autophagy via redox regulation and Sirt1 expression
Source: Cell Death Dis. 2015 Jul 16;6(7):e1827–. doi: 10.1038/cddis.2015.193 (PMC4650738; doi:10.1038/cddis.2015.193)
Supplement: Supplementary Figures [file cddis2015193x1.ppt]

## Slide 1
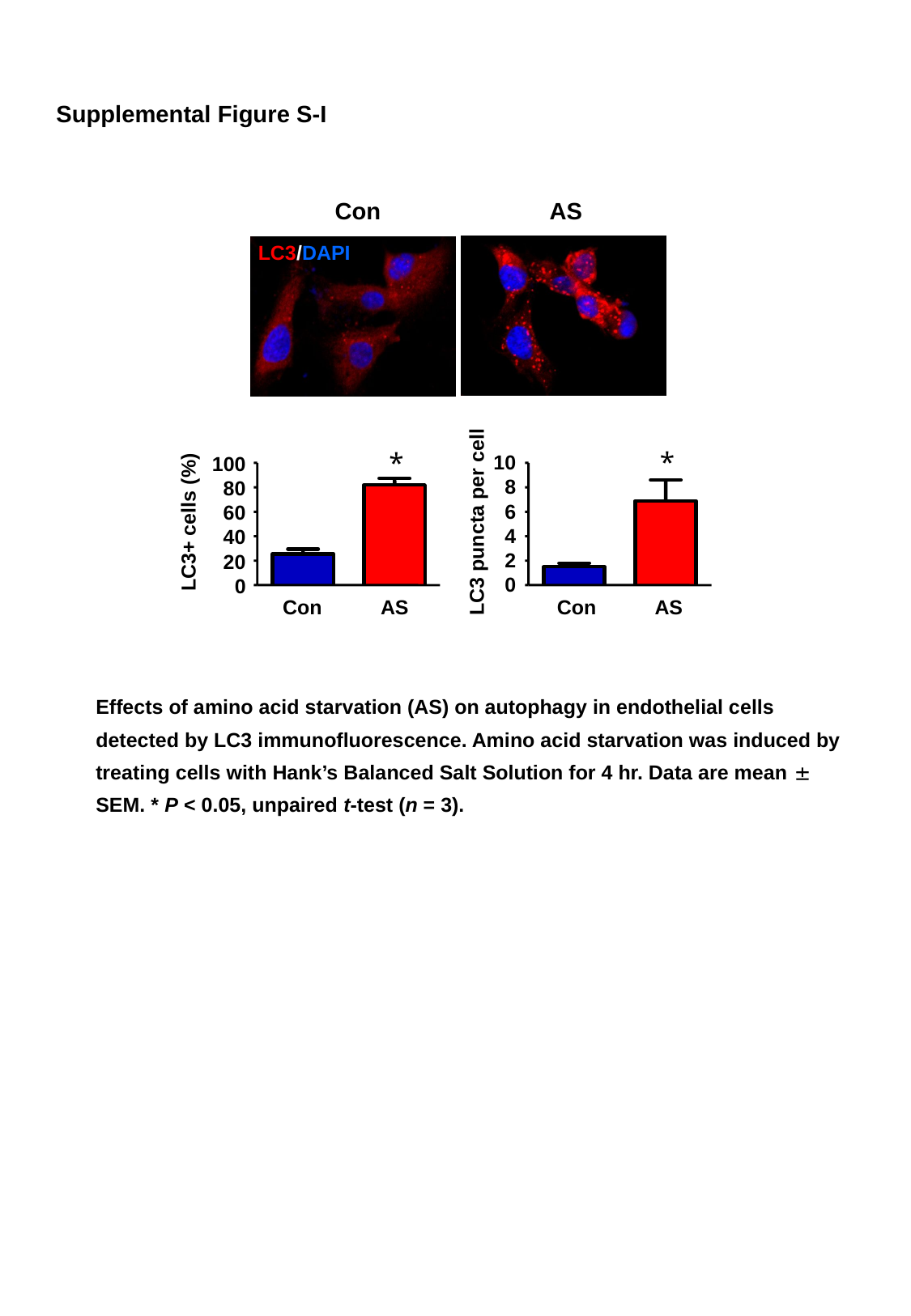

Supplemental Figure S-I
Con
AS
LC3/DAPI
*
*
10
100
8
80
6
60
LC3 puncta per cell
LC3+ cells (%)
4
40
2
20
0
0
Con
AS
Con
AS
Effects of amino acid starvation (AS) on autophagy in endothelial cells detected by LC3 immunofluorescence. Amino acid starvation was induced by treating cells with Hank’s Balanced Salt Solution for 4 hr. Data are mean ± SEM. * P < 0.05, unpaired t-test (n = 3).

## Slide 2
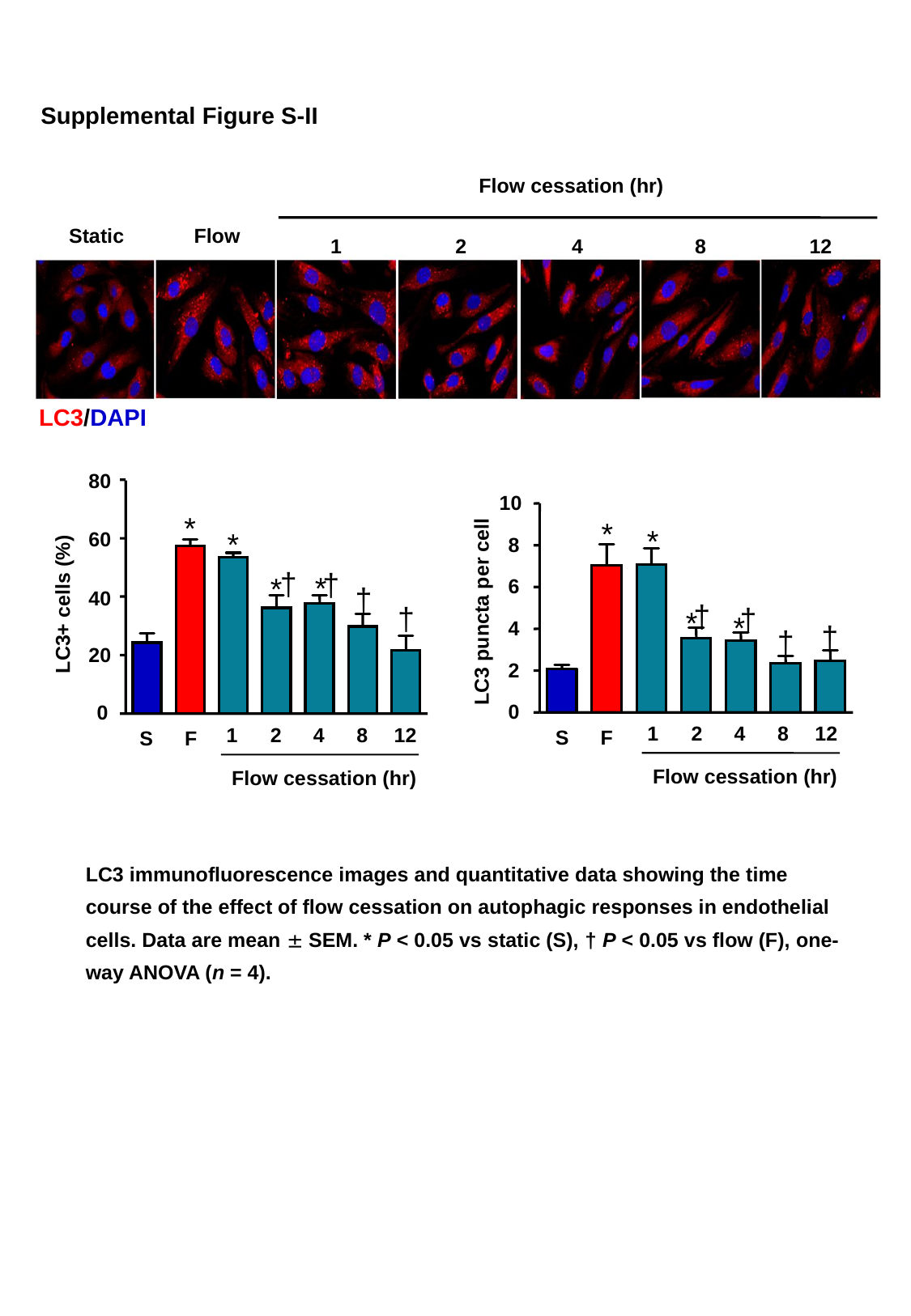

Supplemental Figure S-II
Flow cessation (hr)
Static
Flow
1
2
4
8
12
LC3/DAPI
80
10
*
*
*
*
60
8
†
†
*
*
†
6
40
†
LC3+ cells (%)
†
†
*
LC3 puncta per cell
*
†
4
†
20
2
0
0
1
2
4
8
12
1
2
4
8
12
S
F
S
F
Flow cessation (hr)
Flow cessation (hr)
LC3 immunofluorescence images and quantitative data showing the time course of the effect of flow cessation on autophagic responses in endothelial cells. Data are mean ± SEM. * P < 0.05 vs static (S), † P < 0.05 vs flow (F), one-way ANOVA (n = 4).

## Slide 3
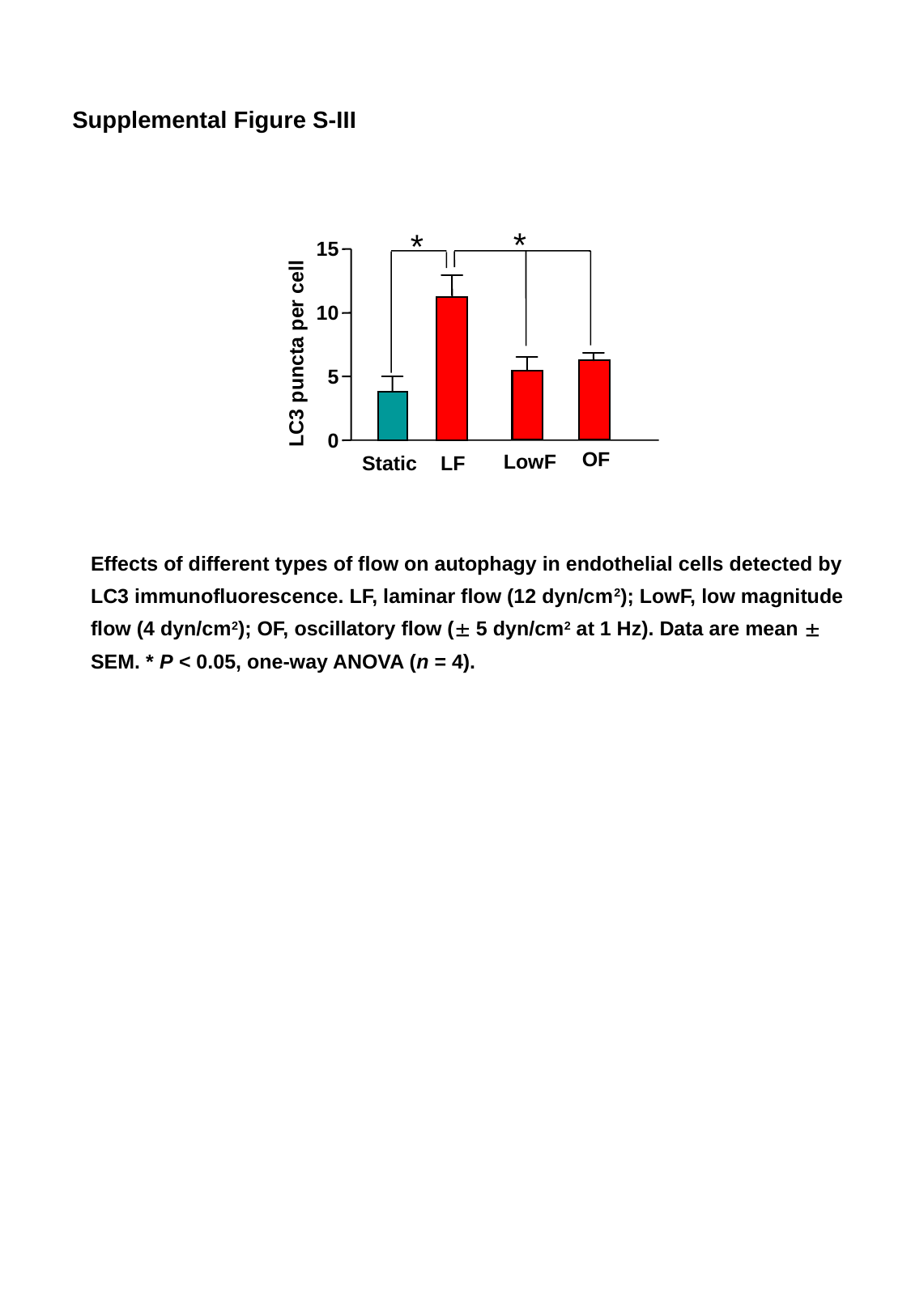

Supplemental Figure S-III
*
*
15
10
LC3 puncta per cell
5
0
OF
LowF
Static
LF
Effects of different types of flow on autophagy in endothelial cells detected by LC3 immunofluorescence. LF, laminar flow (12 dyn/cm2); LowF, low magnitude flow (4 dyn/cm2); OF, oscillatory flow (± 5 dyn/cm2 at 1 Hz). Data are mean ± SEM. * P < 0.05, one-way ANOVA (n = 4).

## Slide 4
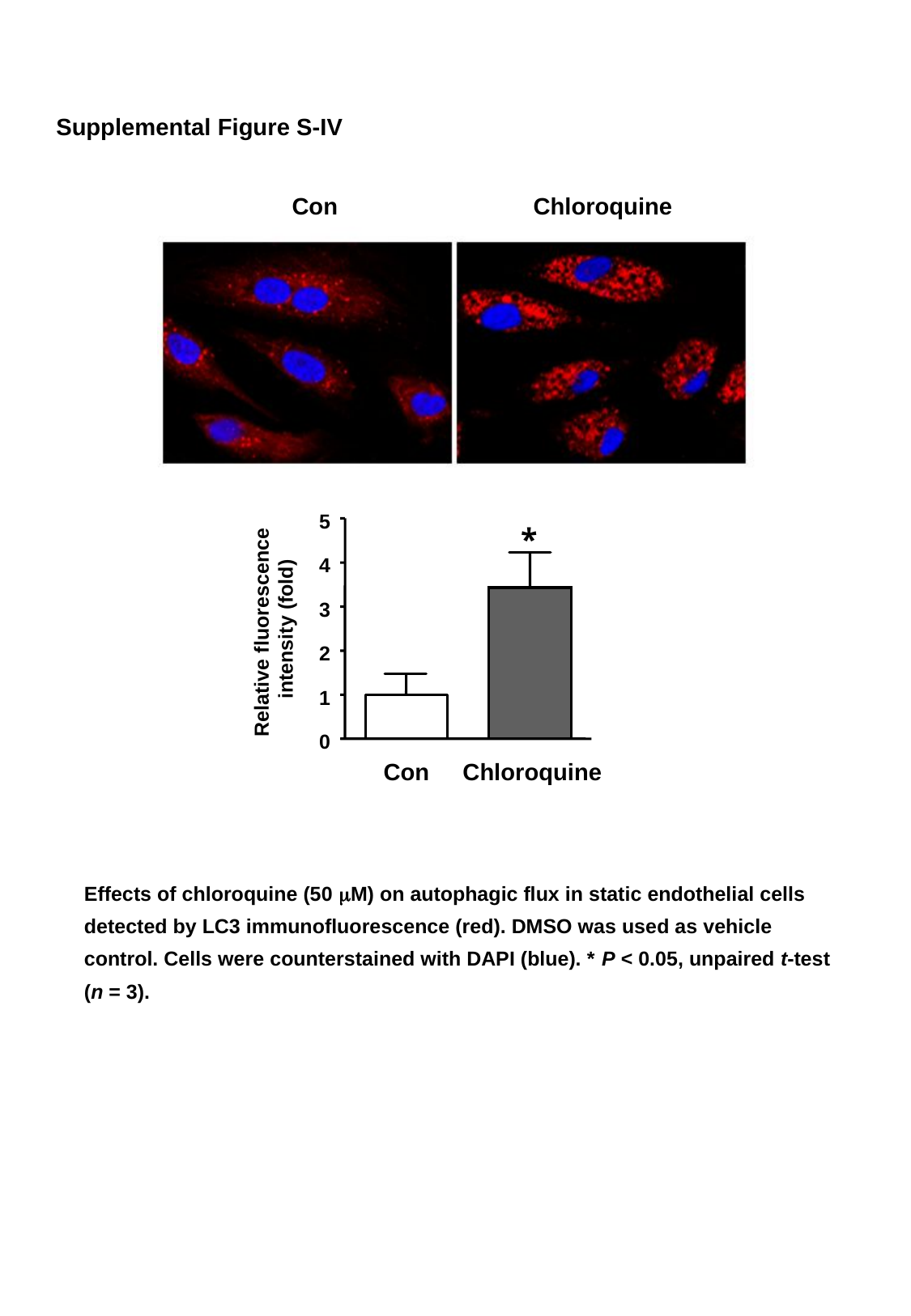

Supplemental Figure S-IV
Con
Chloroquine
5
*
4
3
Relative fluorescence
intensity (fold)
2
1
0
Con
Chloroquine
Effects of chloroquine (50 μM) on autophagic flux in static endothelial cells detected by LC3 immunofluorescence (red). DMSO was used as vehicle control. Cells were counterstained with DAPI (blue). * P < 0.05, unpaired t-test (n = 3).

## Slide 5
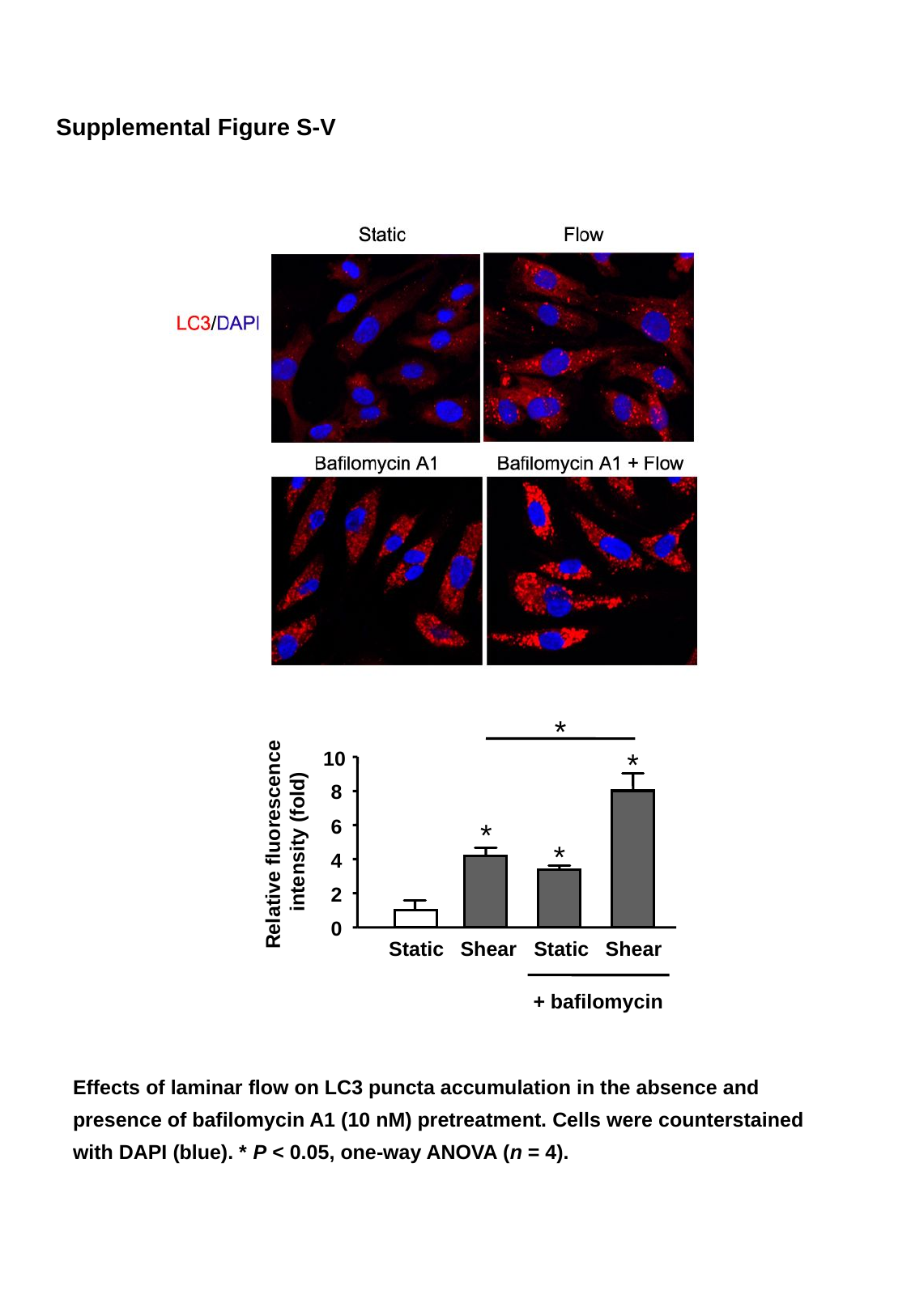

Supplemental Figure S-V
*
*
10
8
*
6
Relative fluorescence
intensity (fold)
*
4
2
0
Static
Shear
Static
Shear
+ bafilomycin
Effects of laminar flow on LC3 puncta accumulation in the absence and presence of bafilomycin A1 (10 nM) pretreatment. Cells were counterstained with DAPI (blue). * P < 0.05, one-way ANOVA (n = 4).

## Slide 6
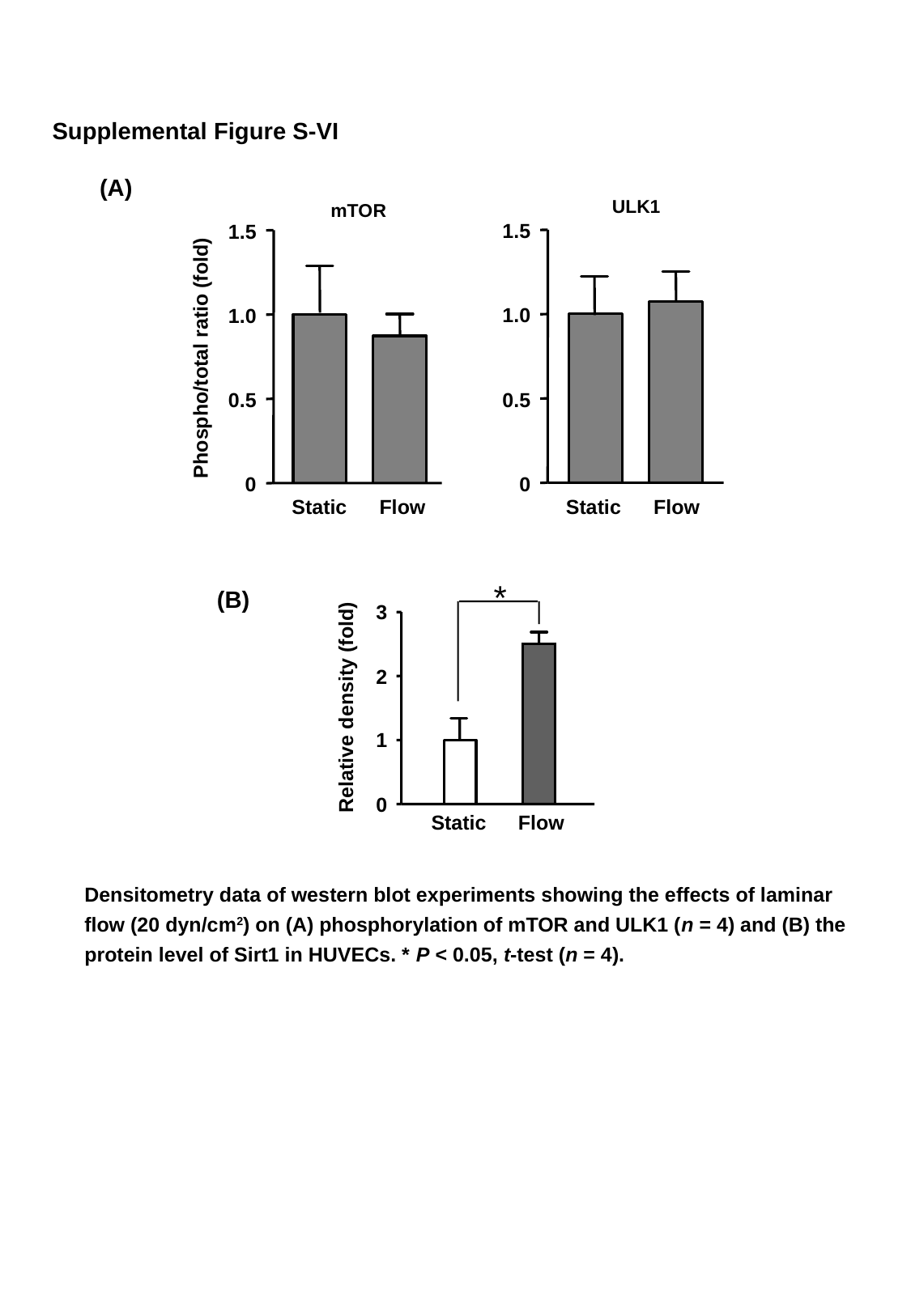

Supplemental Figure S-VI
(A)
ULK1
mTOR
1.5
1.5
1.0
1.0
Phospho/total ratio (fold)
0.5
0.5
0
0
Static
Flow
Static
Flow
*
(B)
3
2
Relative density (fold)
1
0
Static
Flow
Densitometry data of western blot experiments showing the effects of laminar flow (20 dyn/cm2) on (A) phosphorylation of mTOR and ULK1 (n = 4) and (B) the protein level of Sirt1 in HUVECs. * P < 0.05, t-test (n = 4).

## Slide 7
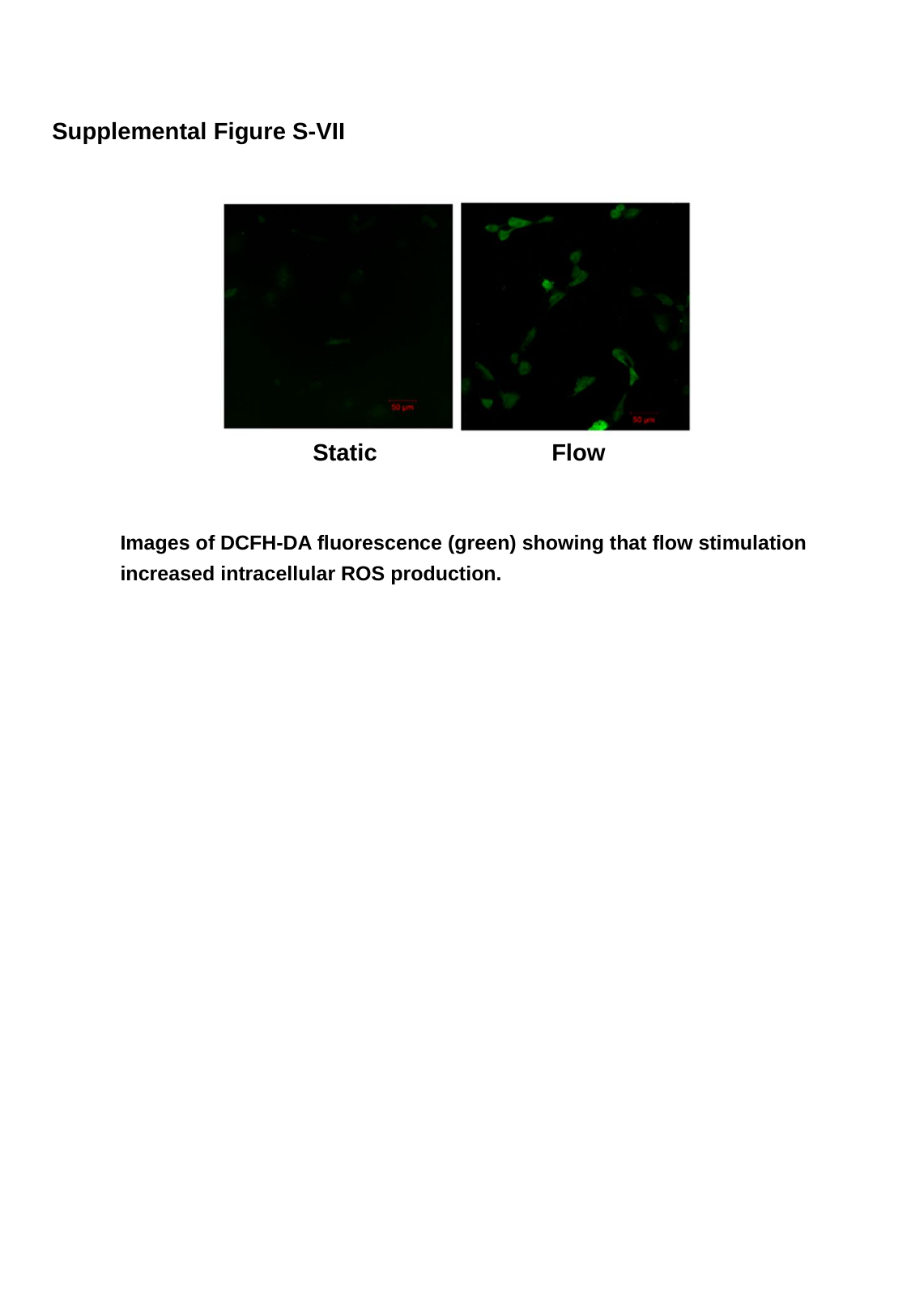

Supplemental Figure S-VII
Static
Flow
Images of DCFH-DA fluorescence (green) showing that flow stimulation increased intracellular ROS production.

## Slide 8
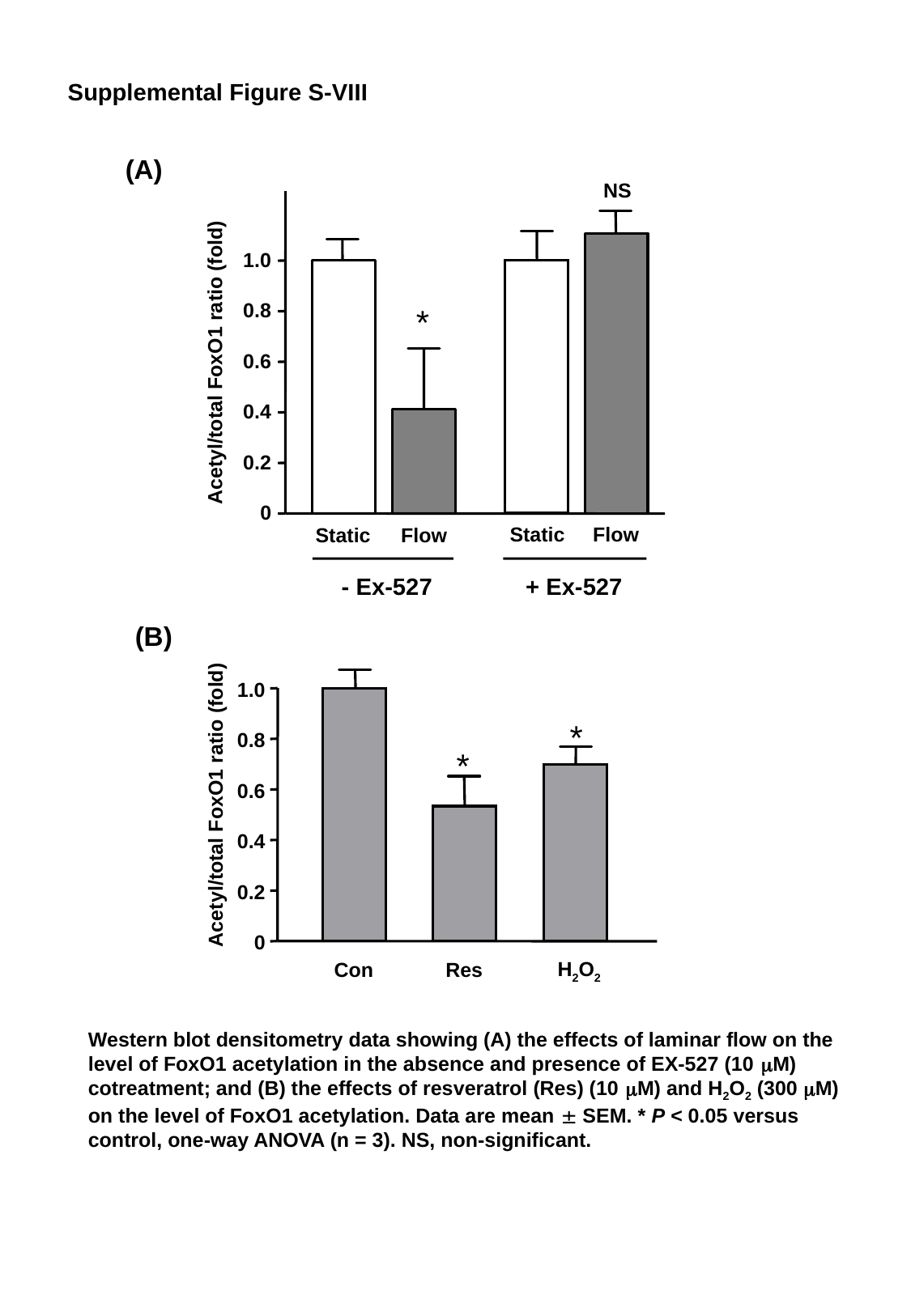

Supplemental Figure S-VIII
(A)
NS
1.0
*
0.8
Acetyl/total FoxO1 ratio (fold)
0.6
0.4
0.2
0
Static
Flow
Static
Flow
- Ex-527
+ Ex-527
(B)
1.0
*
0.8
*
0.6
Acetyl/total FoxO1 ratio (fold)
0.4
0.2
0
H2O2
Con
Res
Western blot densitometry data showing (A) the effects of laminar flow on the level of FoxO1 acetylation in the absence and presence of EX-527 (10 μM) cotreatment; and (B) the effects of resveratrol (Res) (10 μM) and H2O2 (300 μM) on the level of FoxO1 acetylation. Data are mean ± SEM. * P < 0.05 versus control, one-way ANOVA (n = 3). NS, non-significant.

## Slide 9
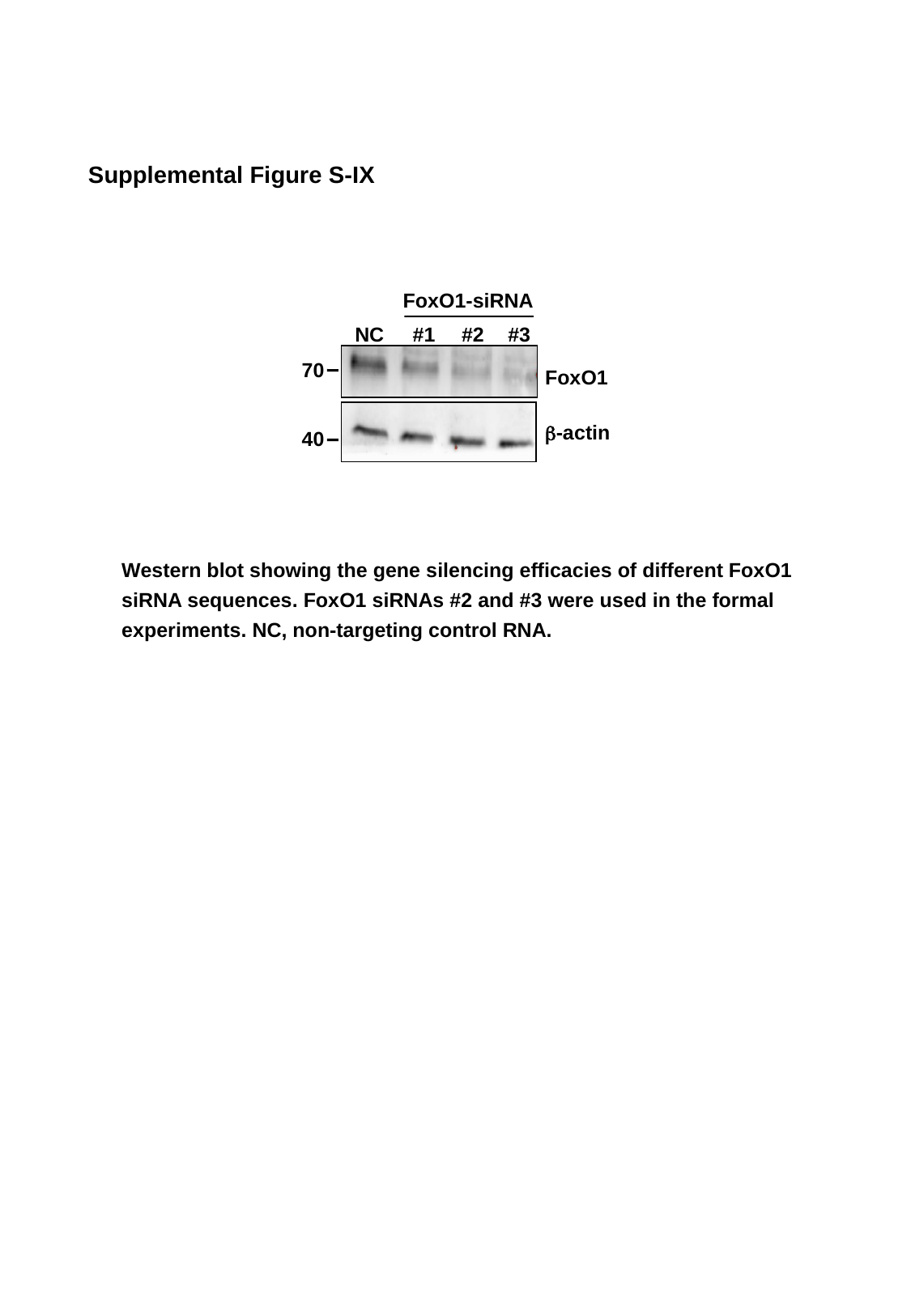

Supplemental Figure S-IX
FoxO1-siRNA
NC
#1
#2
#3
70
FoxO1
β-actin
40
Western blot showing the gene silencing efficacies of different FoxO1 siRNA sequences. FoxO1 siRNAs #2 and #3 were used in the formal experiments. NC, non-targeting control RNA.

## Slide 10
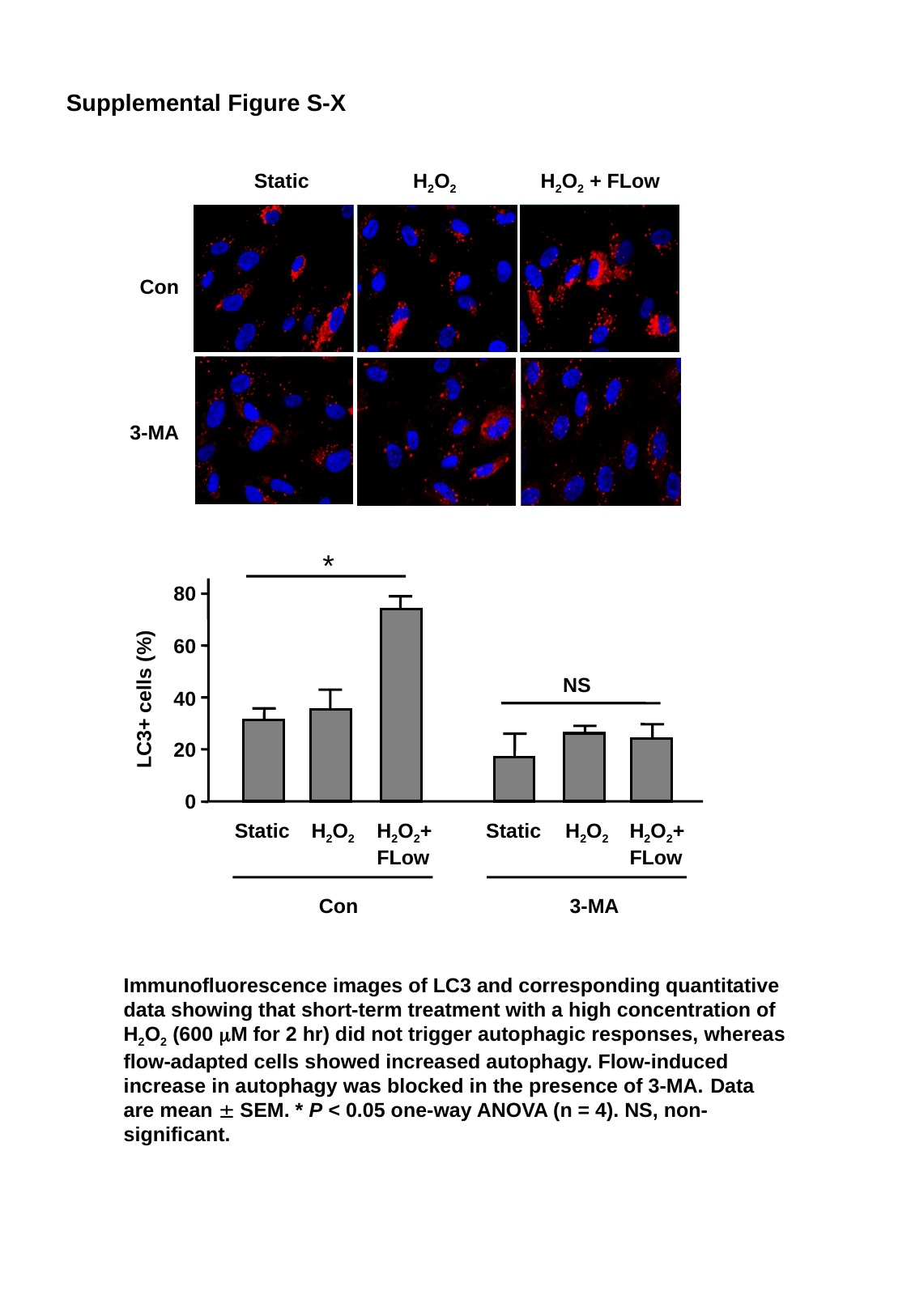

Supplemental Figure S-X
Static
H2O2
H2O2 + FLow
Con
3-MA
*
80
60
NS
LC3+ cells (%)
40
20
0
Static
H2O2
H2O2+
FLow
Static
H2O2
H2O2+
FLow
Con
3-MA
Immunofluorescence images of LC3 and corresponding quantitative data showing that short-term treatment with a high concentration of H2O2 (600 μM for 2 hr) did not trigger autophagic responses, whereas flow-adapted cells showed increased autophagy. Flow-induced increase in autophagy was blocked in the presence of 3-MA. Data are mean ± SEM. * P < 0.05 one-way ANOVA (n = 4). NS, non-significant.

## Slide 11
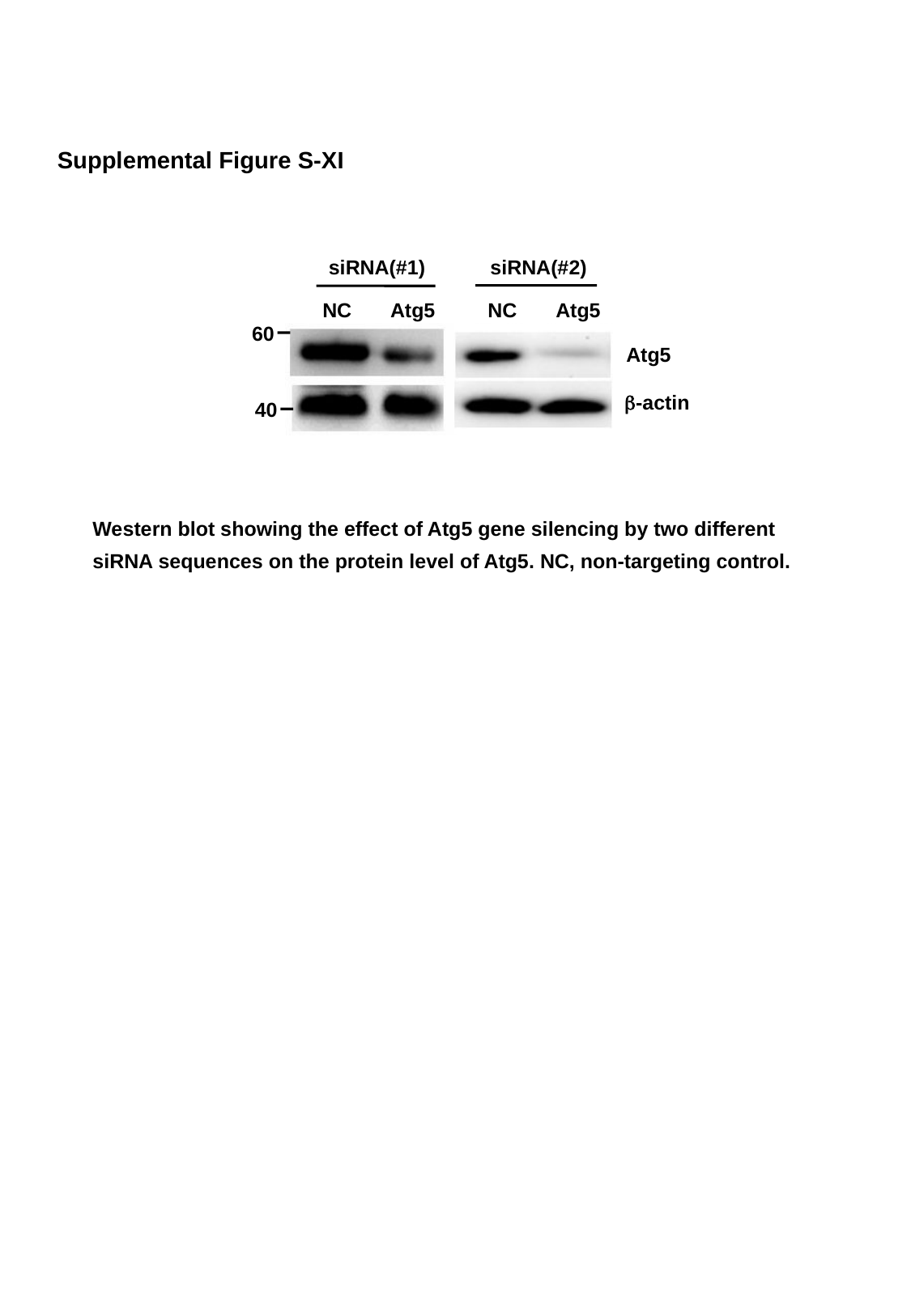

Supplemental Figure S-XI
siRNA(#2)
siRNA(#1)
NC
Atg5
NC
Atg5
60
Atg5
β-actin
40
Western blot showing the effect of Atg5 gene silencing by two different siRNA sequences on the protein level of Atg5. NC, non-targeting control.
